# RED light promotes flavonoid and phenolic accumulation in *Cichorium* spp. callus culture as anti-candida agent

**DOI:** 10.1038/s41598-024-85099-0

**Published:** 2025-01-16

**Authors:** Sayeda Abdelrazek Abdelhamid, Alaa I. Marzouk, Mohsen S. Asker, Hattem M. El Shabrawi

**Affiliations:** 1 Microbial Biotechnology Department, National Research Centre, Dokki, Cairo, 12311 Egypt; 2 Plant Biotechnology Department, Biotechnology Research Institute, National Research Centre, Dokki, Cairo, 12311 Egypt

**Keywords:** *Cichorium endive* supp. *Pumillum*, *Candida*, Monochromatic lights, Flavonoids, Phenolics, Plant biotechnology, Biotechnology, Microbiology, Biological techniques

## Abstract

Chicory species, particularly *Cichorium endive* Supp. *Pumillum*, also, known as Egyptian chicory, are globally recognized for their rich content of bioactive secondary metabolites such as flavonoids and phenolics. These metabolites are highly valued for their pharmaceutical, dietary, and commercial applications. Light exposure, particularly through red and blue wavelengths, is a potent natural elicitor that influences the biosynthesis of secondary metabolites and impacts plant morphology. This study investigates the effects of red and blue LED light exposure on the callus culture of Egyptian chicory (*Cichorium endive* Supp. *Pumillum*), with the aim of enhancing flavonoid accumulation for potential use as an anti-*Candida* agent. Callus cultures of *Cichorium intybus*, *Cichorium endive* Supp. *Pumillum*, and *Taraxacum officinale* (Italian chicory) were grown on MS media supplemented with 4 mg/L 2iP and 0.5 mg/L NAA for 4 weeks. The cultures were then exposed to 12 days of red and blue LED light. After extraction using liquid nitrogen and methanol, the resulting callus extracts were tested against *Candida albicans* NRRL477 at various concentrations (1/8, 1/4, and 1/2 MIC) for 20 to 120 min. The antifungal activity was assessed by determining the effects on acid-soluble phosphorus, total lipids, and soluble proteins in the *Candida* cells. Our results demonstrate that the red LED light-exposed *Cichorium endive* Supp. *Pumillum* callus extract exhibited the most potent antifungal activity, significantly inhibiting the growth of *Candida* species compared to blue light and control treatments. Notably, the red light-treated callus culture accumulated higher concentrations of flavonoids and phenolic compounds, which contributed to its effectiveness as an anti-*Candida* agent. These findings suggest that LED red light elicitation is an effective method for enhancing the production of bioactive compounds in Egyptian chicory, offering potential for its use in natural antifungal therapies. Future research will explore the mechanistic pathways of flavonoid accumulation under different light conditions and investigate the broader applications of this elicitation technique for other medicinal plants.

**Supplementary Information:**

The online version contains supplementary material available at 10.1038/s41598-024-85099-0.

## Introduction

The creation of high-quality pharmaceuticals that are both safe and efficient is one of the pharmaceutical industry’s top priorities. Such medications are produced using plant ingredients, specifically medicinal plants. It contributes significantly to the expansion of the range of medical items. According to the World Health Organization (WHO) prediction and the WHO Traditional Medicine Strategy 2014–2023, in 15–20 years, the share of herbal medicines in the total range of medications might climb to 60%^1^.

*Candida* strains, predominantly *Candida albicans*, are opportunistic pathogens that can cause a range of infections in humans, from superficial mucosal infections to life-threatening systemic diseases, particularly in immunocompromised individuals^2^. The increasing prevalence of drug-resistant *Candida* species has led to a growing interest in exploring alternative treatments, including plant extracts, due to their potential efficacy and safety profiles. Plant extracts have been utilized for centuries in traditional medicine for their diverse pharmacological properties, including antimicrobial activity. These extracts contain a myriad of bioactive compounds, such as polyphenols, alkaloids, flavonoids, and terpenoids, which exhibit various biological activities, including anti-*Candida* effects^3^. Their multi-targeted action may mitigate the development of resistance, making them promising candidates for combating *Candida* infections. In recent years, research into plant extracts as potential anti-*Candida* agents has intensified, with numerous studies demonstrating their inhibitory effects against different *Candida* species. These extracts have shown efficacy in inhibiting *Candida* growth, biofilm formation, and virulence factors, while also, possessing synergistic effects when used in combination with conventional antifungal drugs. Moreover, plant extracts offer several advantages over conventional antifungal agents, including broader spectra of activity, lower cytotoxicity to mammalian cells, and the potential for topical application. Additionally, the abundance of plant sources worldwide provides a rich pool of natural compounds for exploration, offering a sustainable and environmentally friendly alternative to synthetic drugs^4,5^.

Fungi, one of the most significant eukaryotic kingdoms, demonstrate diverse life cycle patterns^6^. More than 6.5 million progressive infections are caused by fungal disorders annually, and around 25% of these infections are related to the *Candida* genus^7^. A commensal yeast prevalent in the human gut microbiome, *Candida albicans*, may turn pathogenic and result in potentially fatal systemic infections, or candidiasis^8^. According to^9^, long-term broad-spectrum antibiotic treatment, invasive surgical procedures, cancer treatment, immunosuppression, and chemotherapy are some of the predisposing circumstances that might lead to invasive candidiasis.

Because of its high dietary fiber content, chicory is a significant crop. Chicory taproots include intriguing secondary metabolite molecules with bioactive qualities in addition to inulin. Differentiated plant cell cultures known as hairy roots have proven to be viable biotechnological hosts for the synthesis of a variety of chemicals originating from plants. Chicory cultivars were identified, and an evaluation was conducted on their potential as a source of antibacterial compounds. It was demonstrated that although similar chicory taproots had negligible action, hot water extracts of hairy roots exhibited antibacterial activity against pertinent human pathogens. The hot water extracts of chicory Callus Culture exhibited remarkable antibacterial activity against methicillin-resistant Staphylococcus aureus, suggesting that chicory extract has great potential to be used as a host for the synthesis of antimicrobial compounds^10^. Plant secondary metabolites (PSMs) have been shown to have a wide range of biological actions that benefit human health. PSM’s popularity is because of their distinguishing characteristics, and they now account for a sizable percentage of the pharmaceutical sector. Obtaining secondary metabolites from wild plants has significant downsides, including time, risk of species extinction from over-exploitation, and restricted production. Plant tissue culture techniques are being used to produce essential secondary metabolites in vitro, marking a paradigm change. Elicitation appears to be a promising strategy for boosting the phytochemical content and quality of medicinal plants. In vitro, culture elicitation triggers the plant’s defense response and enhances the synthesis of secondary metabolites in greater quantities, which is beneficial for medicinal purposes. In this regard, light has emerged as a novel and effective elicitor for increasing the in vitro generation of pharmacologically significant secondary metabolites^11^.

In vitro, cultures of numerous plant species have previously been shown to elicit secondary metabolites under a variety of light conditions. This evaluation divides these sources into three major categories: UV lights, LED lights, and fluorescent lights, which are compared and analyzed in length. Compared to the other UV groups, UV-C radiation is the most effective for stimulating the development of plant secondary metabolites such as phenolics, alkaloids, and flavonoids. The amount of light a plant receives greatly influences its development, growth, and productivity. Agriculture uses typical artificial light sources including high-pressure sodium lamps (HPSLs), metal-halide lamps (MHLs), and fluorescent lamps (FLs) to create a controlled environment. Among these, fluorescent lights have become more popular. Nevertheless, these lighting sources’ wavelengths range from 350 to 750 nm, which is regarded as low quality for plant growth and development. Their low photosynthetic flux and limited lifespan of activity make them unsuitable for use in plant illumination systems that demand high agricultural productivity. The introduction of new kinds of semiconductor materials has allowed LED technology to be used in an increasing number of new fields, such as plant growth and development. LEDs have been shown to be a more intelligent artificial lighting source than conventional lighting systems for creating controlled environments in vitro and agricultural systems. LEDs can be used to control the quantity of photosynthetically active and photomorphogenic radiation needed for plant growth and development since they emit light within specific spectral ranges^12–14^.

Reference^1^ explore how artificial light and specific plant growth regulators influence both cell growth and secondary metabolite production, specifically inulin. Their findings highlight that controlled light conditions, especially those incorporating specific wavelengths, can significantly enhance the biosynthesis of valuable secondary metabolites in chicory, thus supporting the medicinal potential of this plant species. This aligns closely with our work, which examines the use of LED light as an elicitor to stimulate the production of secondary metabolites in chicory callus cultures. By applying red and blue LED light to the callus culture of Egyptian chicory, we aim to investigate and optimize the elicitation of flavonoids and phenolic compounds with potential antifungal properties. Kirakosyan^1^ results reinforce the relevance of light manipulation in controlled plant tissue culture systems to boost the production of bioactive compounds, which is central to our objective of developing effective anti-*Candida* agents through enhanced secondary metabolite synthesis.

Given the rising interest in natural compounds with antifungal properties, particularly against pathogens like *Candida albicans*, there is a need to explore sustainable methods for enhancing the production of bioactive compounds in medicinal plants. Egyptian chicory (*Cichorium endive* subsp. *pumillum*) holds significant promise due to its rich profile of flavonoids and phenolic compounds. This study aims to investigate the effects of specific light wavelengths, namely red and blue LED light, as physical elicitors on the callus cultures of Egyptian chicory. By stimulating secondary metabolite production, particularly flavonoids, we seek to optimize chicory extracts as potential anti-*Candida* agents. Our objective is to determine how red and blue light exposure impacts phytochemical accumulation and antifungal efficacy, ultimately supporting the development of enhanced natural antifungal products.

## Materials and methods

### Place of study

This study was carried out in Plant Biotechnology Department, In collaboration with Microbial Biotechnology Department, National Research Center (NRC), Giza, Egypt.

### Plant materials

#### Seed collection

In vitro callus of three chicory species *Cichorium intybus*, *Cichorium endive* Supp. *Pumillum* and *Taraxacum officinal*e had been examined to two light colors (Red and Blue) for 12 days continuously along with the normal color as control. After they had been extracted using liquid nitrogen and Methanol as each 1 g of elicited callus dissolved in 10 ml MeOH, same done for the intact plant of each species. In total 12 samples had been collected to examined on HPLC^15^. The seed of both *Cichorium intybus* and *Taraxacum officinale* had beed bought online from Amazon.com in 2020, however *Cichorium endive* Supp. *Pumillum* seed has been brought from the agriculture research center, Giza, Egypt, in 2020.All work on sterilization of seeds by using 20% hypo chloride sodium (NaOCl) for 20 min, then washed by distilled sterilized water 4–5 times, seedlings obtained on the 5th day from germination time. After surface disinfection, seeds were cultivated on a PGR-free nutrient medium of Murashige and Skoog (MS). The pH of the medium was adjusted to 5.6–5.8 before being autoclaved at 121 °C and 1.1 atm for 20 min. The cultures were maintained in a culture room at 25 ± 2 °C during a long-day photoperiod (16 h of light: 8 h of dark) with cool white fluorescent light (2000–2500 lx)^15^.

#### Callus induction

The callus culture of chicory plant species was initiated from leaf explant on MS media containing 4 mg/L 2iP and 0.5 mg/L NAA for 4 weeks, The pH of the medium was adjusted to 5.6–5.8 before being autoclaved at 121 °C and 1.1 atm for 20 min. The cultures were maintained in a culture room at 25 ± 2 °C during a long-day photoperiod (16 h of light: 8 h of dark) with cool white fluorescent light (2000–2500 lx)^15^. After the callus transferred to a new media and elucidated under different light quality for varied time periods^15^.

#### Antimicrobial activity

The antimicrobial activities were carried out according to the conventional agar diffusion test^16^ using cultures of *Bacillus subtilis* NRRL B-94, *Pseudomonas aeruginosa* NRRL, and *Candida albicans* NRRL477. The bacterial strains were cultured on nutrient medium, while the yeast strains were cultured on malt medium and yeast medium, respectively. Broth media included the same contents of nutrient medium except for agar. For bacteria and yeast, the broth media were incubated for 24 h, 0.5 mL of inocula were added to 50 mL of agar media (50 °C) and mixed by simple inversion. The agar was poured into 120-mm Petri dishes and allowed to cool to room temperature. Wells (9 mm in diameter) were cut in the agar. Wells were filled to the surface of agar with the plant extract to be tested. The plates were left for 1 h at refrigerator to allow the diffusion of plant extract, and then plates were incubated at 37 °C for 24 h. The inhibition zone appeared was measured with a ruler.

### Minimal inhibitory concentration (MIC) determination

Determination of the MIC for 3 species of Candida (*Candida rogusa* ATCC 10571, *Candida albicans* NRRL477, and *Candida albicans* ATCC10231) were measured by optical density assay for the different microbial strains by added different volumes of different plant extracts at 5 mL nutrient broth culture was then inoculated with 50 µL of the different *Candida* fresh cultures and incubated in shaking incubator at 120 rpm at 37 °C for 24 h. Microbial growth was measured at 620 nm, and the results were expressed as growth inhibition percentage.

### Mode of action

The effects of different concentrations of the *Cichorium endive* Supp. *Pumillum* red and *Cichorium endive* Supp. *Pumillum* blue on some biochemical activities were studied. Immediately after incubating flasks with *Candida albicans* NRRL477, cells were harvested during the middle logarithmic growth phase, and plant extracts were applied in concentrations of 1/8, 1/4 and 1/2 MIC. Each test was repeated three times. Subsequently, the flasks were shaken using a rotary shaker of 120 rpm at 37 °C. Samples were withdrawn at onset of the experiment and after incubation periods of 20, 40, 60, 80, 100 and 120 min. The *Candida* cells were subjected to the following determinations: acid soluble phosphorus compounds, total lipids, and soluble protein.

### Extraction of intercellular components of *Candida* cells

#### Acid-soluble phosphorous compounds

Acid-soluble phosphorous was determined according to^17,18^. The cells were collected by centrifugation, washed twice with ice cold saline and extracted twice with 5% ice cold trichloroacetic acid (TCA). The suspensions were finally, centrifuged at 5,000 rpm. The extract (1 mL) was added to 4 mL reagent (40 mL of 6 N H2SO4, 80 mL distilled water, 40 mL ammonium molybdate solution and 40 mL ascorbic acid), mixed and incubated at 37 °C for 2 h and then cooled at room temperature. The absorbance was measured at 680 nm.

#### Total lipid

Total lipid was determined according to^19,20^. The residue after removing of the acid-soluble compounds was extracted three times with a mixture of chloroform– methanol (2:1, v/v). A volume of 0.1 mL of extract was added to 5 mL of concentrated H2SO4. The mixture was heated for 10 min in a boiling water bath and cooled, and 0.4 mL aliquot was placed in a dry test tube. Six milliliters of phosphor-vanillin reagent (0.6 gm vanillin dissolved in 10 mL ethanol before diluting to 100 mL with distilled water was mixed with 400 mL of concentrated orthophosphoric acid) was then added to each test tube. The mixture was set in the dark for 45 min, and the absorbance was measured at 525 nm.

#### Soluble protein

Soluble protein was determined according to^21^. The delipidated cells were solubilized in 1 N KOH at 37 °C for 20 h. The protein in the extract was determined at 595 nm.

### Statistical analysis

All experiments were performed in triplicate, and the mean values are presented. The results were given as mean ± standard deviation and ANOVA. Statistical analyzes were performed using IBM SPSS Statistics 20.

## Results

**Fig. 1:**
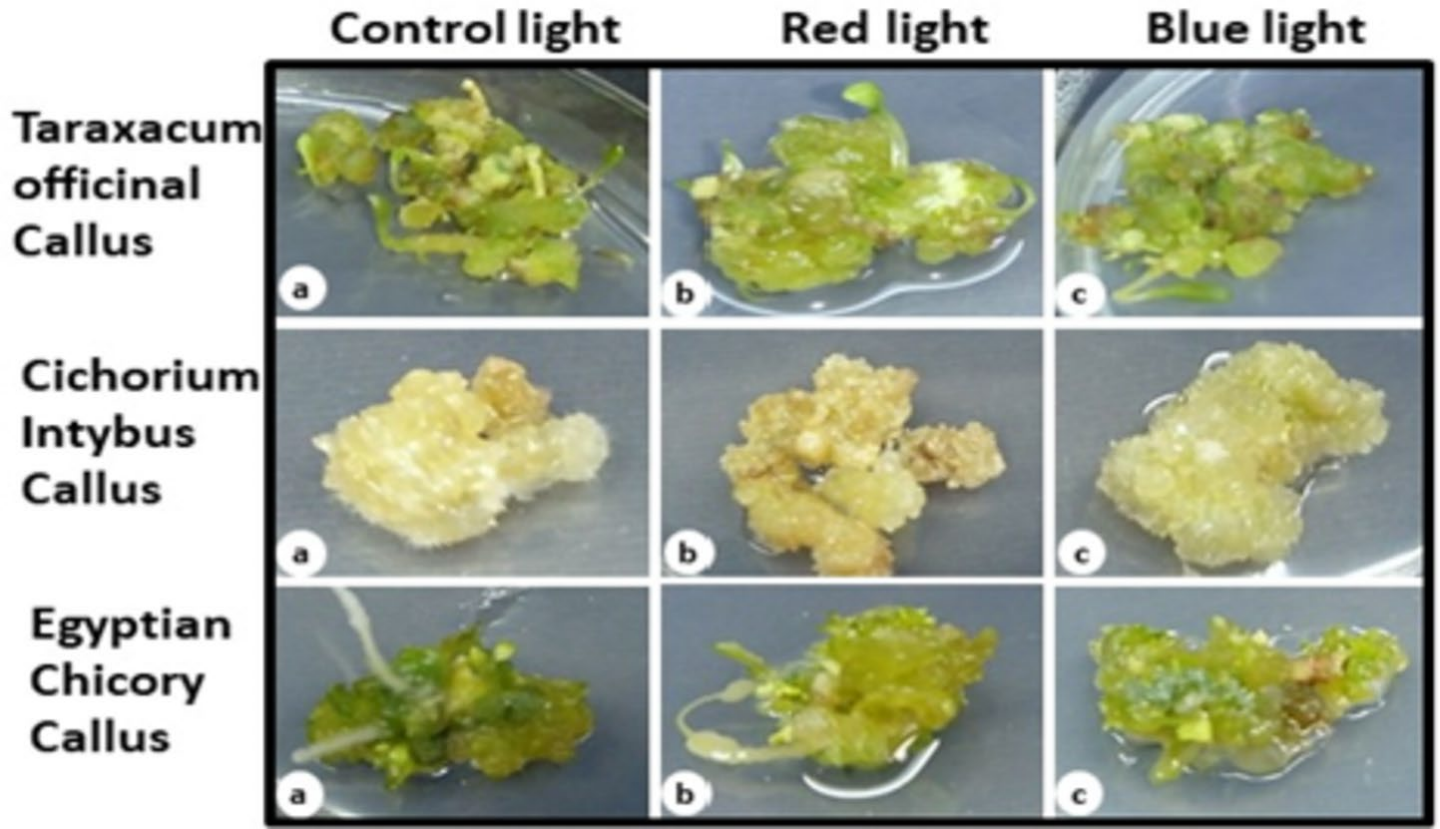
The Chicory species callus culture after exposed to different color light for continuance 15 days. The panel (**a–c**) for each chicory cultivar indicate to the normal, Red and Blue color treatment during callus in vitro growth.

The callus culture of chicory plant species was initiated on MS media containing 4 mg/L 2iP and 0.5 mg/L NAA for 4 weeks. The callus structure and proliferation morphological characterization was different between three species *C. Intybus*,* C. endive* Supp. *Pumillum* (Egyptian chicory) and *Taraxacum officinale* (Dandelion) (Italian chicory) depending on the plant species and the balance of different wavelengths present in the light source Fig. 1. The *T. officinal* growth is proliferating like *C. Intybus* Fig. 1 (a, b and c), *C. endive* Supp. *Pumillum* Fig. 1 (a, b and c) friable green callus they are same initiating micro leafs and shoots from callus cells in addition to the rapid growth rate of their culture in the main while and under the same conditions of different light treatments the *C. Intybus* Fig. 1 (a, b and c) whitish compact callus growth with no kind of proliferation at all and less growth rate than other chicory species. However, the differences in exposing the chicory callus culture to light color the figure did not show any significant difference between them. Note that these cultures of chicory species are growing under same MS media composition for callus induction purpose.

**Fig. 2:**
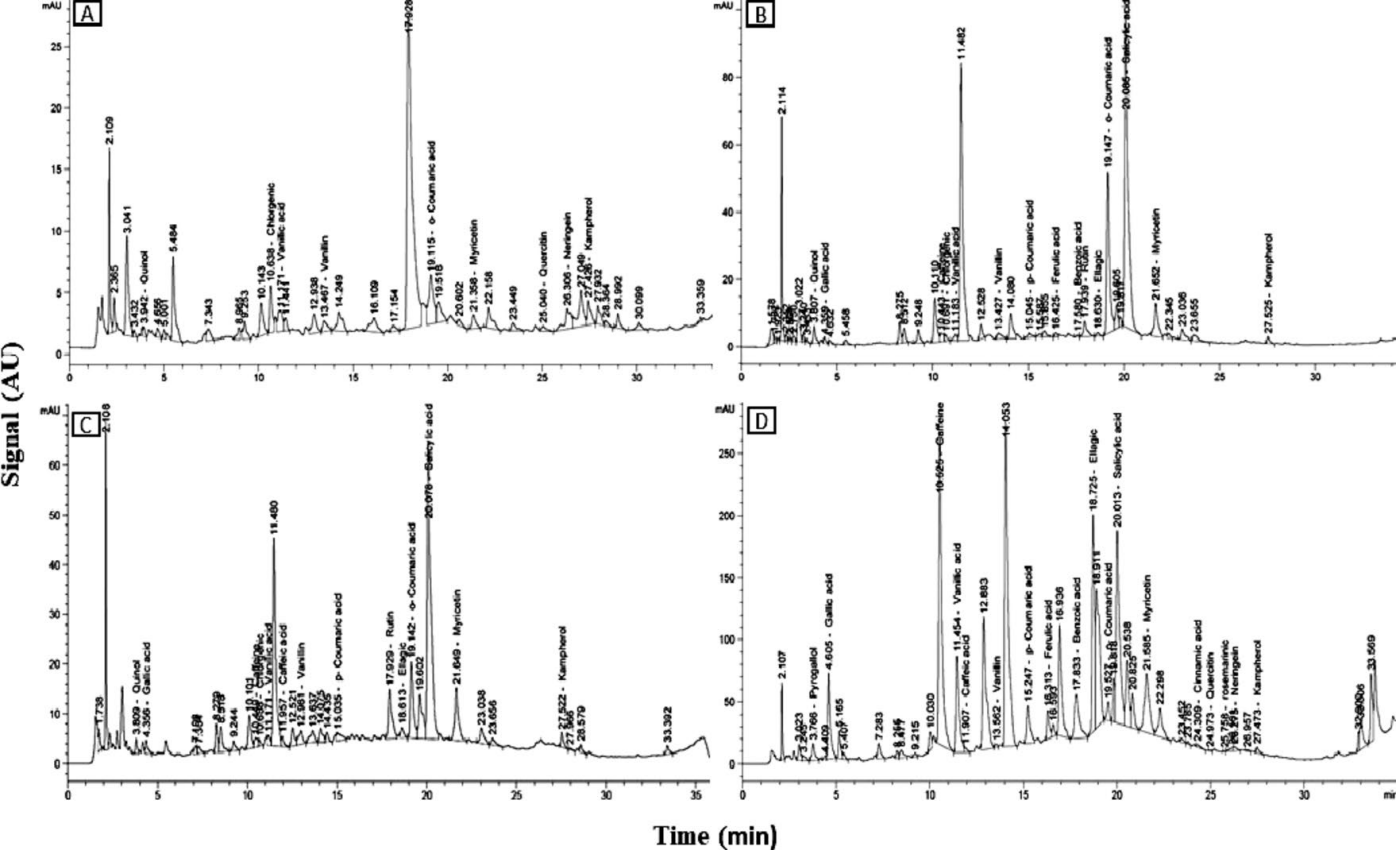
HPLC-UV chromatogram of a intact plant and callus methanol extract of *C. endive* supp. Pumilum “Egyptian chicory” exposed to (normal, blue and red lights)showed in (**B–D**) comparing to intact plant (**A**) targeted phenolic compounds under optimal gradient elution conditions at 280 nm. Peaks identification as in Table 1.

Table 1; Fig. 2 shows the flavonoids and phenolic compounds concentrations of the elucidated callus cultural of *Cichorium endive* Supp. *Pumillum* to red and blue lights separated by HPLC in µg/ml. the HPLC value indicated to that red light could accumulate more flavonoids and phenolic compounds than the blue and control light. The myricetin 1648 ug/ml and kamapherol 61, 42 ug/ml were the highest accumulated compound under red light elicitation, while the rutin and quinol were accumulated only under red color. The flavonoids compounds neringein and rosmarinic were not detected under the blue light. In the other hand the phenolic compounds accumulation detected values under fed light were more interested since the data showed several phenolic compounds couldn’t be detected in control or blue light and existed only with red light with high concentration by the HPLC detector. For example, gallic, salyslic acid, caffien, and benzoic acid (6702.71, 1200.34, 1112.5 and 640.83 ug/ml) respectively were detected with very high amount comparing to the control and blue light. “Also”, the pyrogallol, ferulic acid and cinnamic acid were detected only under red light, while the benzoic acid and caffeic acid could not be detected under blue light. These data concluded that the physical red light elicitation can be a critical factor to promote and accumulate phenolic and flavonoids compounds of Egyptian chicory callus culture.

**Table 1:** The phytochemical constituents of elicited callus extract of *Cichorium Endive* supp. Pumillum with Redand Blue Light by HPLC.

No.	Flavonoids	Concentration under normal light (µg/ml)	Concentration under red light (µg/ml)	Concentration under blue light (µg/ml)
1	Myricetin	277.5	1648	313.52
2	Neringein	00	84. 5	00
3	Kamapherol	20.03	61.42	29.32
4	Rosmarinic	9.32	9.32	00
5	Vanillin	1.75	1.80	3.67
6	Rutin	35.74	00	86.53
7	Quinol	14.34	00	7.61

No.	Phenolics	Concentration under normal light (µg/ml)	Concentration under red light (µg/ml)	Concentration under blue light (µg/ml)
1	o-Coumaric A.	37.78	8.31	11.81
2	p-Coumaric A.	1.11	28.52	8.91
3	Caffeine	4.399	640.83	00
4	Vanillic acid	3.066	210.54	3.182
5	Gallic Acid	1.951	106.362	3.067
6	Pyrogallol	00	30.73	00
7	Ellagic	23.77	6702.71	89.79
8	Ferulic acid	00	26.13	00
9	Cinnamic acid	00	1.17	00
10	Salicylic acid	653.43	1200.34	479.14
11	Caffeic acid	00	1.66	2.23
12	Benzoic A.	8.83	1112.5	00
13	Chlorogenic acid	2.23	00	1.4

Table 2 showed the dimensions of the inhibition zones of antimicrobial activity of some chicory species on different microorganism growth using well diffusion methods.

**Table 2:** Showed the dimensions of the inhibition zones of antimicrobial activity of some chicory species on different microorganism growth using well diffusion methods.

Plant extract	Bacillus subtilis NRRL B-94	Pseudomonas aeruginosa NRRL	Candida albicans NRRL477
	Inhibition zone (mm)		
*Cichorium endive* (control)	0.00	0.00	15.00
*Cichorium endive* (red)	0.00	0.00	30.00
*Cichorium endive* (blue)	0.00	0.00	25.00
*Cichorium intybus* (control)	23.00	0.00	14.00
*Cichorium intybus* (red)	30.00	0.00	20.00
*Cichorium intybus* (blue)	26.00	0.00	18.00
*Taraxacum officinal* (control)	0.00	0.00	12.00
*Taraxacum officinal* (red)	0.00	0.00	16.00
*Taraxacum officinal* (blue)	0.00	0.00	15.00

Data showed the specificity of the Egyptian chicory “*Cichorium endive* Supp. *Pumillum*” to inhibit the growth of *Candida* cells through high numbers of inhibition zone diameter comparing to other *Cichorium* species *C. intybus* or *T. officinal* under red and blue light colors (25 and 30 mm) respectively. “Also”, data revealed that there is no effect at all on *Pseudomonas aeruginosa* NRRL growth from all *Cichorium* species, while only *C. intybus* chicory shoed good effect and large inhibition zone for the *Bacillus subtilis* NRRL B-94 nothing else. This result could indicate the specificity of the Egyptian chicory to inhibit the *Candida* cell growth.

### The minimal inhibitory concentration (MIC)

**Fig. 3:**
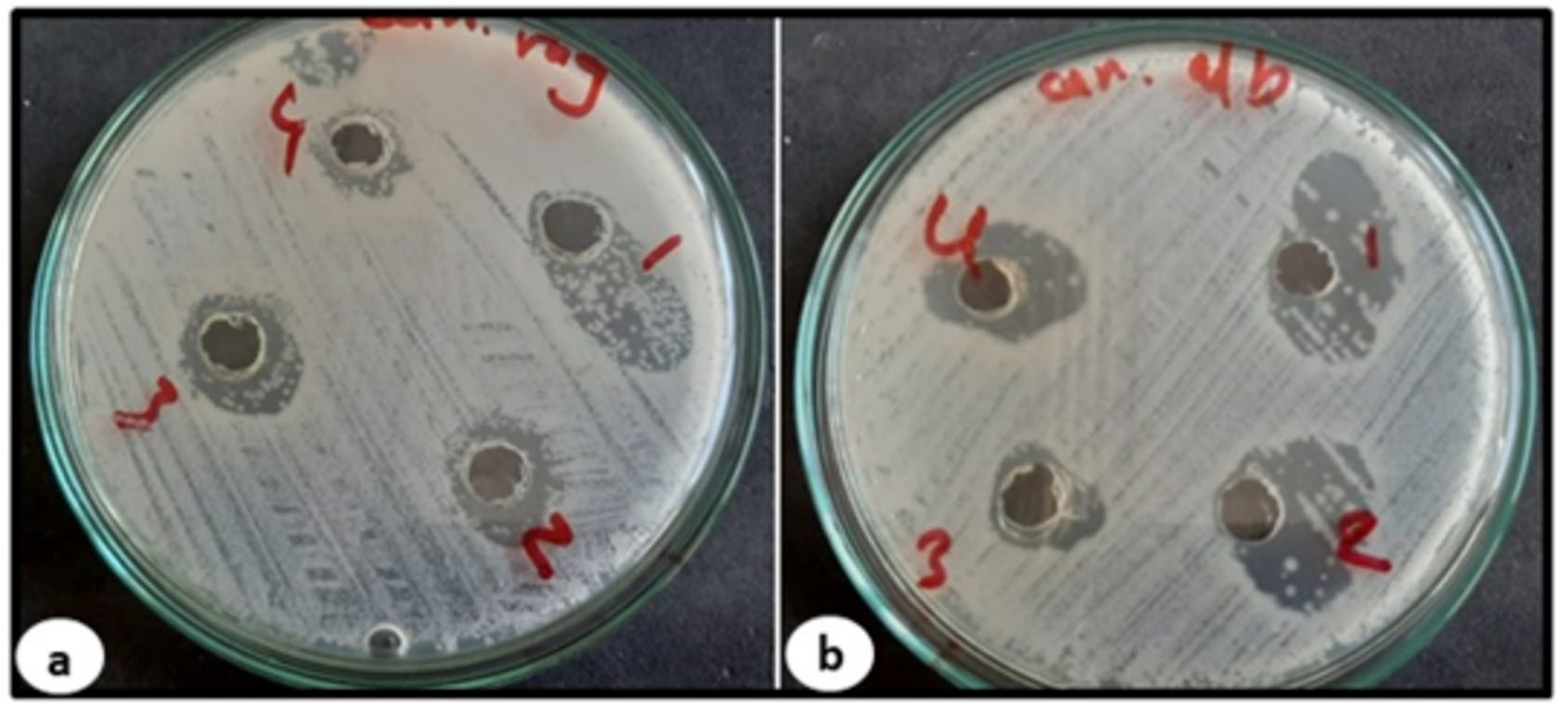
The Clear zone inhibition of *Candid regusa* (**a**) and *Candida albicans* (**b**) growth in yeast media after adding different callus chicory extracts concentrations for 24 h in agar diffusion test plat.

The MIC activity values of *Cichorium endive Supp. Pumillum* callus culture extract treated with physical elicitation of color lights and exposed to *Candida* yeast cells. The red light elicitation treatment showed the best MIC value especially with *C. albican.* NRRL477 comparing to normal and blue color light for all *Candida* species. However, *Cichorium endive Supp. Pumillum* red with MIC activity values ranging from 0.25 to 2 µg, and *Cichorium endive Supp. Pumillum* blue with MIC activity values from 50 to 100 µg Table 3; Fig. 3. Minimum inhibitory concentration (MIC) of different plant extracts for various plan species extracts on different strains of *Candida*. In contrast, the aqueous extract of chicory callus exhibited significant ant-*Candida* effects, with the best MIC (0.28 ug/mL) value. Moreover, the MICs for chicory callus exposed to normal light as a control against the tested *Candida* were the same (0.45 mg/mL). The negative controls also, did not show any inhibitory effects against the tested *Candida* strains. Based on the findings, among the tested bacteria and yeast strains, *C. albica* (NRRL477) most sensitive to C. endive supp.*Pumillum* extracts, while C. regusa was the most resistant yeast against them.

**Table 3:** The minimal inhibitory concentration (MIC) for the four plants extracts *Cichorium endive supp.* Pumillum control, red and blue with a concentration of 1.50 µg/ml approximately respectively against different candida strains.

Plant extract	Candida rogusa ATCC 10,571	Candida albican NRRL477	Candida albican ATCC10231
	MIC inhibition zone (µg/ml)		
*C. endive Supp.* Pumillum cont.	0.45	0.45	0.45
*C. Supp.* Pumillum *endive* red	0.57	0.28	0.57
*C. Supp.* Pumillum *endive* blue	1.79	0.89	0.89

**Fig. 4:**
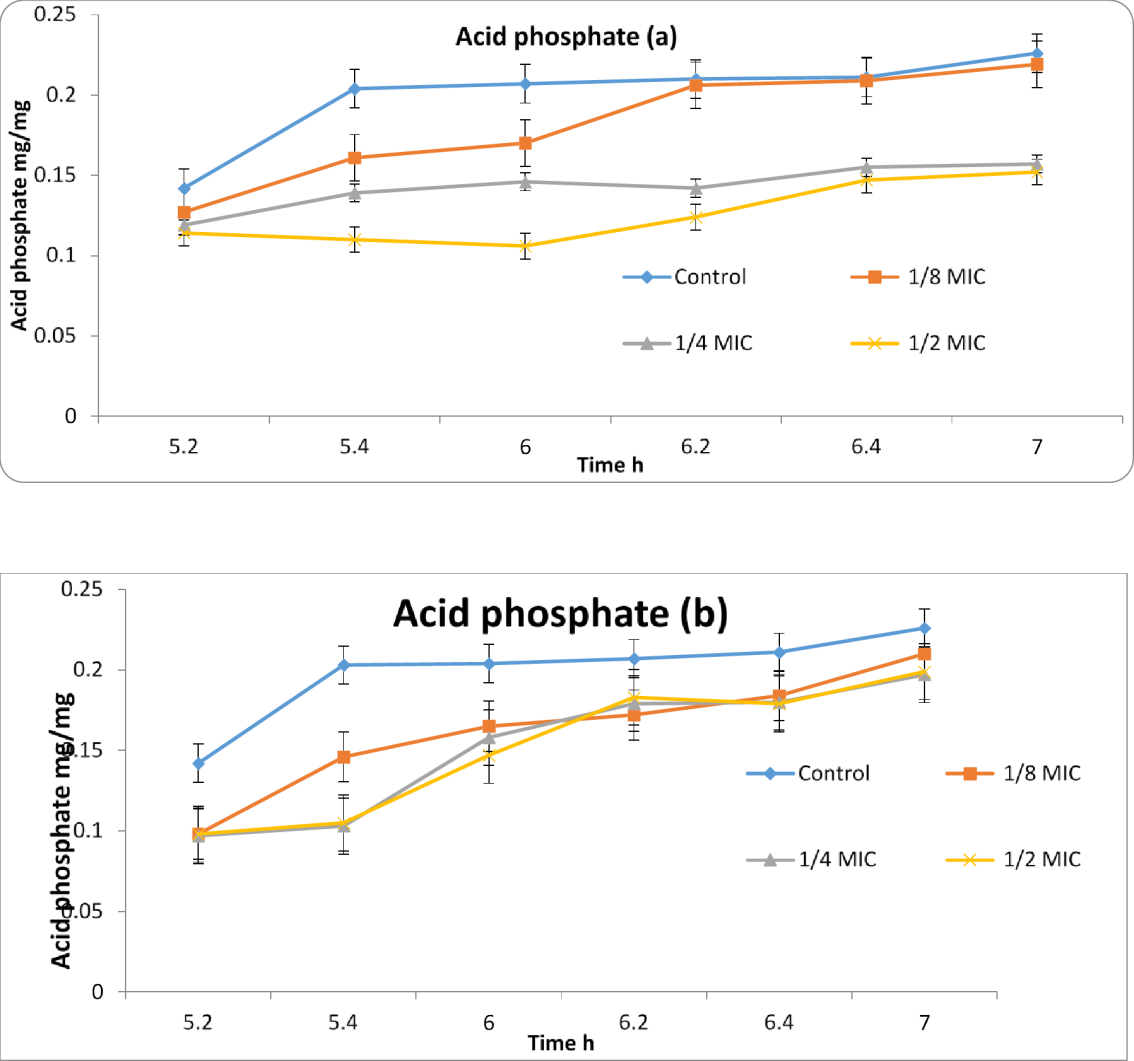
Effect of different concentrations of *C. endive Supp.* Pumillum red (**a**) and *C. endive Supp.* Pumillum blue (**b**) on the biosynthesis of acid-soluble phosphorus in the *Candida albicans*.

**Fig. 5:**
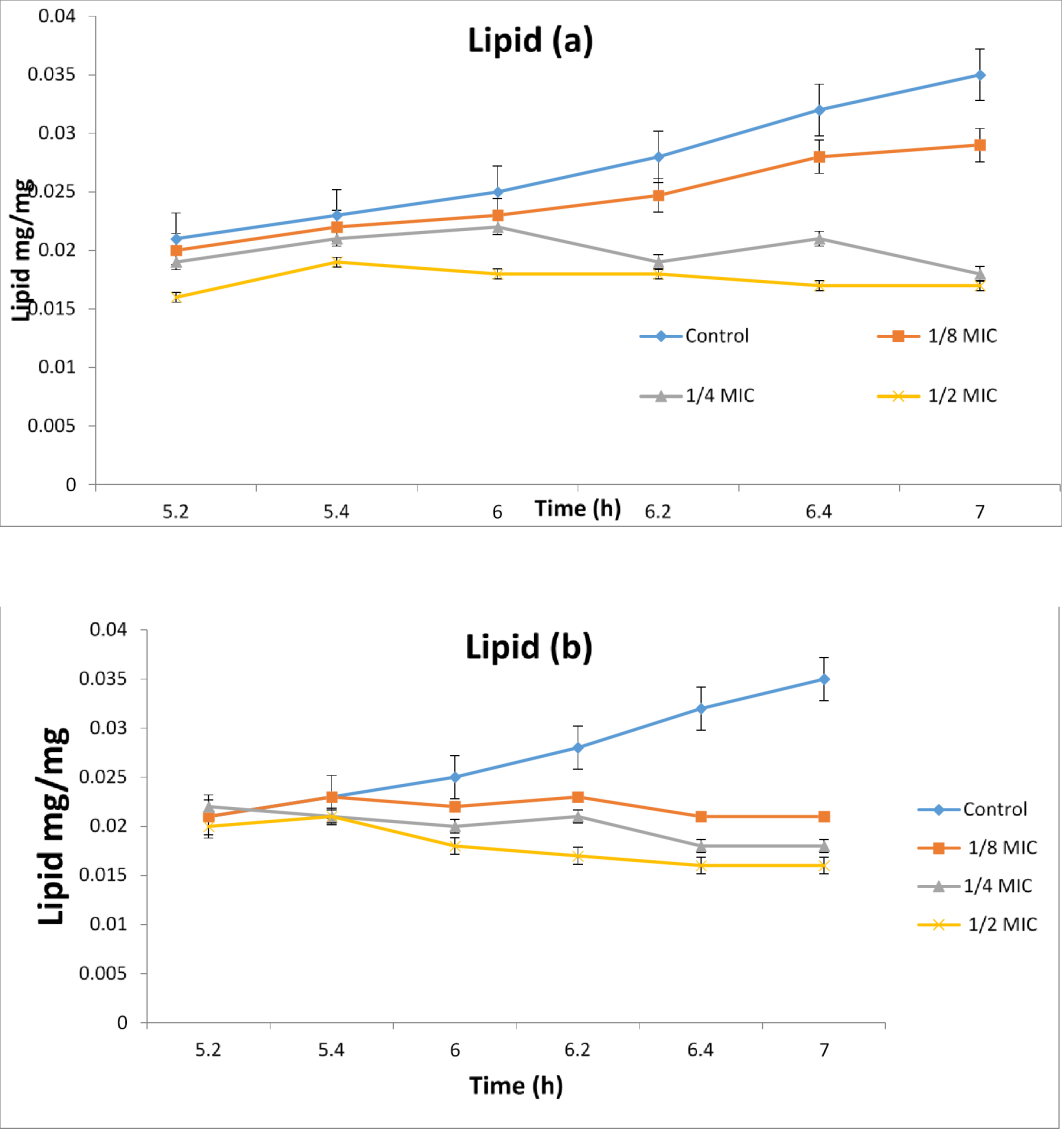
Effect of different concentrations of *C. endive Supp.* Pumillum red (**a**) and *C. endive Supp.* Pumillum blue (**b**) on the biosynthesis of total lipid in the *Candida albicans*.

**Fig. 6:**
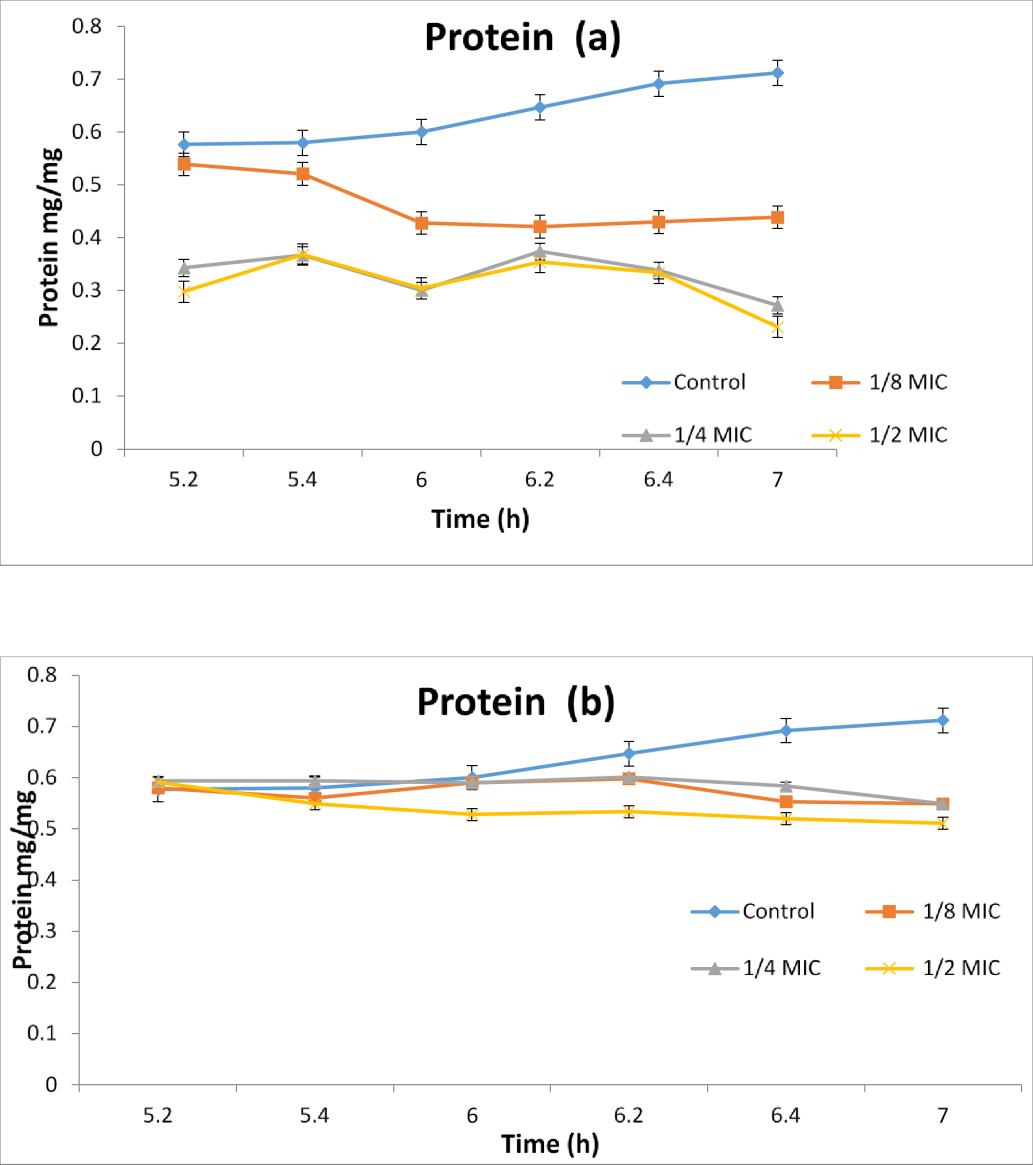
Effect of different concentrations of *C. endive Supp.* Pumillum red (**a**) and *C. endive Supp.* Pumillum blue (**b**) on the biosynthesis of protein in the *Candida albicans*. RED light promotes flavonoid and phenolic accumulation in *Cichorium* spp. callus culture as anti-candida agent.

The effect of plant extracts of the Egyptian callus culture of chicory plant exposed to red and blue light as a physical elicitor compared to the natural light on *Candida albican* contents of acid phosphate which is involved in the structure of *Candida* cell membrane Fig. 4a, b and the effect on the total lipid as well as their effect on the total protein content of *Candida* cells Fig. 5a, b. The results of these basic components of the *Candida* cell membrane showed that the Egyptian chicory callus culture extract did not have a significant effect on the acid phosphate content during the entire experiment period of 120 min, whether exposed to blue or red extract, indicating that the chicory callus culture extract did not have an effect on the components of the *Candida* phospholipids Fig. 5a, b. But if we check the total lipid content values in the four samples (1/8, 1/4 and 1/2 of MIC) of the lethal concentration of *Candida*, we find that they have strongly influenced the fats derived from the *Candida* lipid membrane in the total time period of 120 min, especially the chicory callus red-colored extract compared to the blue and control color. “Finally”, if we look at the total protein values of the *Candida* cells Fig. 5a, b, we will find some vulnerability from the Egyptian chicory callus extract on it, especially the blue-colored extract that clearly shows the differences between the protein values between them at all tested time points from (20 to 120 min) Fig. 6a, b. These data indicate that the Egyptian chicory callus extract and exposed to red light has a strong effect on the total fat analysis of the Candia membrane structure, which is often more present in the composition of cell membranes of the *Candida* yeast growth.

## Discussion

Antifungal prescription drugs that frequently appear are confronted with issues related to human toxicity and inherent or acquired resistance by *Candida* species^22,23^. Finding alternative antifungal treatments that are effective and safe for treating a range of *Candida* infections becomes necessary. In line with research carried out by^24,25^, they noticed that compounds were fungicidal and non-toxic to mammalian cells. The purpose of our current investigation is to find out how they combat *Candida* cells to enhance therapeutic approaches and investigate potential mechanisms of drug interaction with the fungal cell wall, membrane, cellular content, and retreating cytoplasm that cause cell lysis without human toxicity. Chicory extract, derived from the roots of the chicory plant, contains bioactive compounds such as inulin, sesquiterpene lactones, flavonoids and phenolic compounds. These components exhibit various biological activities, including antimicrobial properties^10,26^. Studies have shown that plant extract possesses antifungal activity against *Candida* species by disrupting fungal cell membranes, inhibiting cell growth, and altering cell morphology. Additionally, chicory extract may modulate the host immune response, enhancing the body’s ability to combat fungal infections. Light color elicitation, on the other hand, involves exposing *Candida* cells to specific wavelengths of light, typically in the blue or violet spectrum^27^. Combining chicory extract and light color elicitation may offer synergistic effects against *Candida* growth. Chicory extract could sensitize *Candida* cells to subsequent light exposure, enhancing the efficacy of light-based therapies. Moreover, the antioxidant properties of chicory extract may mitigate cellular damage induced by reactive oxygen species generated during photodynamic therapy, a light-based approach that utilizes photosensitizing agents to induce cell death in microorganisms^28^. All three plants are commercially and commonly utilized as chicory, an edible form. In this study, we conducted a pilot survey of three types of chicory species plants (*Cichorium intybus*, *Cichorium endive* Supp. *Pumillum* “Egyptian chicory” and *Taraxacum officinale* “Italian chicory”) after exposing them to two light colors for continuous 12-day (LED blue and red light) and then there had been extracted using methanol. These extracts were exposed to three types of microorganisms (Bacillus subtilis NRRL B-94, Pseudomonas aeruginosa NRRL, and *Candida albicans* NRRL477), and the Egyptian chicory extract was found to have a major and specialized effect on the *Candida* culture. This extract was then treated with three *Candida* cells. The result showed a significant impact on the Candid cells, especially *Candida albicans* NRRL477 after exposed to red light for 12 continues days, with an effective MIC value. At the time of further study, acid phosphate, total lipid and total protein were extracted from cells that were subjected to dilution from three MIC value cells (1/8, 1/4 and 1/2). The results showed that the Egyptian chicory extract had a strong effect on the composition of the cell membrane after exposer time of 120 min Fig. 5a, b in compare to the acid phosphate that were not affected at all by Fig. 4a, b and the total protein amounts that were relatively affected after the extraction was envisaged for 100 min Fig. 6a, b. When reviewing the chemical composition of the crude chicory extract of flavonoids and phenolics through the results of HPLC concentrations, some phenols found in the Egyptian chicory extract, especially which exposed to LED red light for 12 days, have very high concentrations compared with the exposure to blue light or normal light (e.g., galllic, salyslic acid, caffien, and benzoic acid (6702.71, 1200.34, 1112.5 and 640.83 ug/ml) respectively, and the appearance of compounds (pyrogallol, ferulic acid and cynamic acid) only in the red light extract. Recent research has shown that the majority of these phenol compounds have a significant effect on the *Candida* yeast and the nature of its assembly, affecting the formation of the micro hypha necessary for the formation of microfilm and thus weakening and the formation of cell populations to the host and the event of injury. This also, applies to flavonoids, whose concentrations from the HPLC system have also, led to high concentrations of myricetin 1648 ug/ml and kamavirol 61, 42 ug/ml. The Egyptian chicory extract exposed to red light is also, showed unique compounds such as neringin and rosmarinic compared to other treatments and the cartel^29^. These flavonoid compounds are reported in recent research papers that influence the microfilm of the nature of the growth of the gland reptiles of the *Candida* Nurse’s cells. “Also”, Ref^30^. investigated how myricetin affected the *C. albicans* cells’ membrane permeability, which was measured using assays for intracellular material leakage and crystal violet uptake. He discovered that the proportion of crystal violet uptakes for C. albicans cells treated with myricetin at 1×, 2×, and 4× the myricetin MIC were 36.5, 60.6, and 79.4%, respectively. Their research sheds light on the potential pharmacological value of myricetin as an antifungal therapy for *Candida albicans* infections. Previous report from^31^ reported that ellagic acid is a potential candidate to eliminate *C. albicans*-generated biofilm by suppressing the expression of the involved genes. Ellagic acid inhibited *C. albicans* growth 0.68–82.44%, dose dependently. The determination of the amount of acid phosphate, total lipid, and soluble protein in the treated Candid cells with different dilutions of MIC concentration shows that they became closer to the control after being exposed to chicory extract treated with blue light for 120 min, which means that the blue extract was not very effective on the biosynthesis of the three compounds: acid phosphate, total lipid, and soluble protein. While the chicory extract was exposed to the red light continuously, Easley showed that after 120 min of exposure to *Candida* cells, the amount of acid phosphate, total lipid, and soluble protein decreased compared to the control sample. It means that the composition of phenolic and flavonoids in the extract could have an effect on the biosynthesis of lipid membranes and phospholipid membranes in *Candida* cells, as well as the cell-soluble proteins (Figs. 1, 2 and 3).

The biofilm formation also, reduced 2.61–68.318%. The effects of different light wavelengths, such as red light and blue light, on the accumulation of secondary metabolites like myricetin, ellagic acid, and caffeine in plants can vary depending on the specific plant species, its growth stage, and environmental conditions. The red light has been shown to enhance the accumulation of certain secondary metabolites like anthocyanins and flavonoids, which include myricetin and ellagic acid^32^. This is because these compounds often serve as antioxidants and protective agents in response to environmental stresses^33^. However, Normal white light, which contains a broad spectrum of wavelengths including red, blue, and green, can also, impact secondary metabolite accumulation in plants. The effects of white light may be a combination of the individual effects of its constituent wavelengths. However, the specific response can vary depending on the plant species and the balance of different wavelengths present in the light source.

current study and Kirakosyan^1^, underscore the influence of species, PGR composition, and light quality on chicory callus growth and metabolite accumulation. The findings collectively suggest that while *C. endive* Supp. *Pumillum* is more favorable for applications requiring high phenolic and flavonoid yields.

This study is the beginning of further research to deepen the extent to which exposure to red light is particularly specialized in the accumulation of phenols, flavonoids and possibly other compounds such as Sesquiterpenes, which are often found in the Egyptian chicory plant^10^. The importance of focusing on these types of research that emphasize the use of natural compounds from Egyptian plants, maximizing their production and highlighting their importance and the quality of maximizing the accumulation of such materials in safer and less costly non-industrial ways, such as the use of colorful light.

## Conclusion

In conclusion, our study demonstrates the significant impact of red light exposure on the accumulation of flavonoids and phenolic compounds in *Cichorium endive Supp. Pumillum* (Egyptian chicory) callus culture, showcasing its potential as an effective anti-*Candida* agent. The results indicate that red light elicitation significantly enhanced the concentration of bioactive compounds such as myricetin, kampeferol, gallic acid, and caffeic acid, which were found in higher concentrations compared to blue light or control treatments. These compounds, known for their antimicrobial properties, contributed to the enhanced antifungal activity observed against *Candida albicans*, particularly with the lowest minimum inhibitory concentrations (MIC) in the red light-treated extracts. The red light-exposed chicory extract also, influenced *Candida* cell membrane composition, reducing total lipid and protein content, which is consistent with its antifungal activity.

Our findings highlight the promising application of light-induced elicitation as a non-industrial method to enhance the production of bioactive compounds in plants like Egyptian chicory. The specificity of the Egyptian chicory extract in inhibiting *Candida* growth, particularly in comparison to other species, opens up potential therapeutic uses for treating *Candida* infections, an area that remains critical due to increasing resistance to conventional antifungal treatments. This study lays the groundwork for future research into the synergistic effects of plant extracts and light exposure, with the potential to develop safer, more cost-effective alternatives to current antifungal therapies. Further studies should explore the molecular mechanisms behind these interactions and investigate the broader applicability of red light elicitation in the production of other secondary metabolites with medicinal properties.

## Electronic supplementary material

Below is the link to the electronic supplementary material.

Supplementary Material 1

Supplementary Material 2

Supplementary Material 3

Supplementary Material 4

Supplementary Material 5

Supplementary Material 6

Supplementary Material 7

Supplementary Material 8

Supplementary Material 9

Supplementary Material 10

## Data Availability

All data generated or analysed during this study are included in this published article [all data are here and in supplementary data].
