# Supplementary material for: RED light promotes flavonoid and phenolic accumulation in Cichorium spp. callus culture as anti-candida agent
Source: Sci Rep. 2025 Jan 16;15:2194. doi: 10.1038/s41598-024-85099-0 (PMC11739635; doi:10.1038/s41598-024-85099-0)
Supplement: Supplementary file 1 — Supplementary Material 1 [file 41598_2024_85099_MOESM1_ESM.pdf]

Data File D:\DATA\PHENOLSFLAVONOIDS2019\FSQC487-1801489.D
Sample Name: FSQC487-18

===

Acq. Operator : FSQC Lab
Acq. Instrument : Instrument 1   Location : Vial 1
Injection Date : 10/2/2018 11:16:41 AM
Inj Volume : No inj
Acq. Method : C:\CHEM32\1\METHODS\PHENOLS AND FLAVONOIDS2019NEW_LC.M
Last changed : 10/2/2018 11:08:37 AM by FSQC Lab
(modified after loading)
Analysis Method : C:\CHEM32\1\METHODS\PHENOLS AND FLAVONOIDS2019_MIX_1_LC.M
Last changed : 10/9/2018 10:16:20 AM by FSQC Lab
(modified after loading)
Additional Info : Peak(s) manually integrated

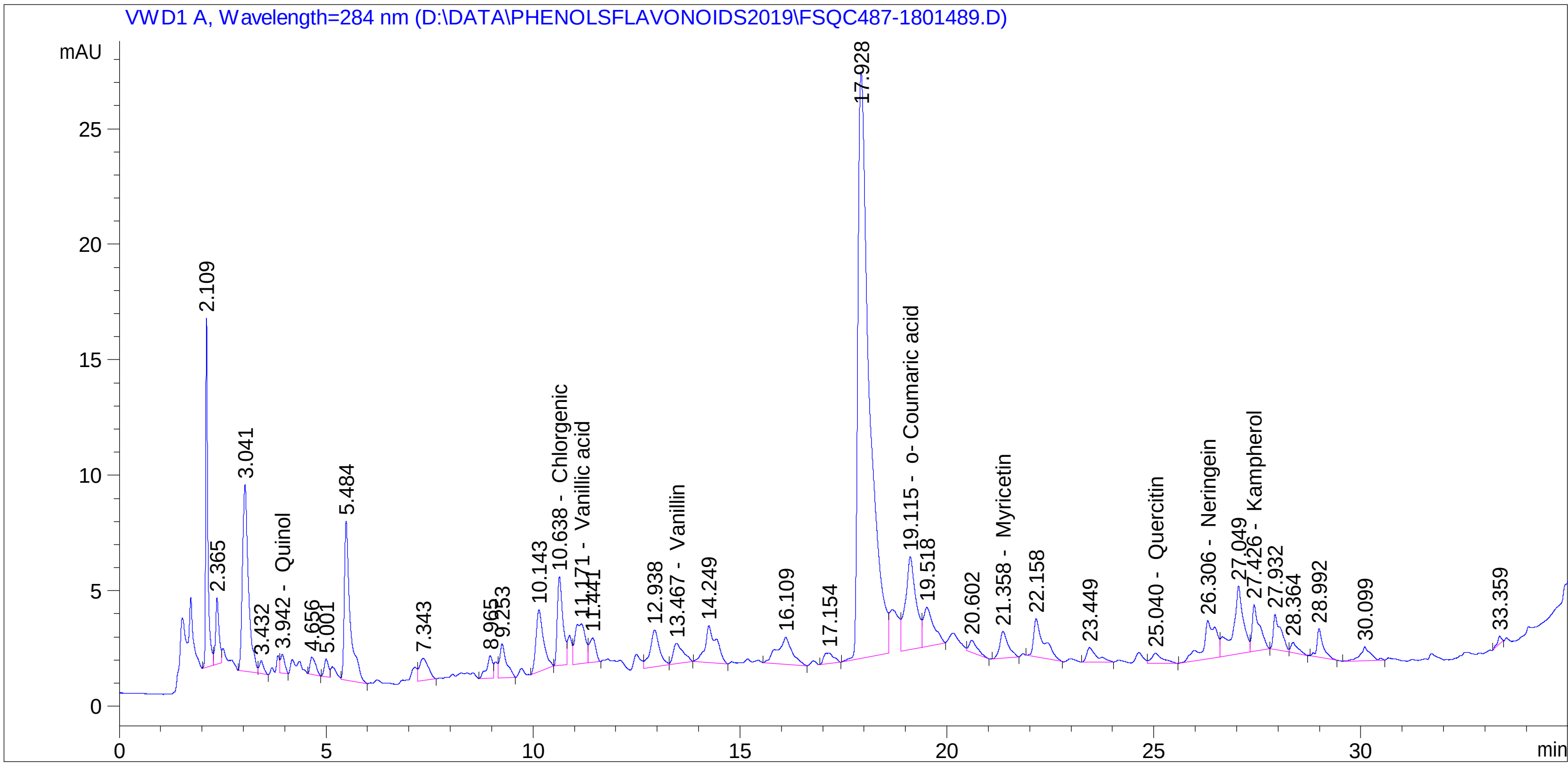

===

External Standard Report

===

Sorted By : Retention Time
Calib. Data Modified : 10/9/2018 10:16:09 AM
Multiplier: : 15.0000
Dilution: : 1.0000
Do not use Multiplier & Dilution Factor with ISTDs

Signal 1: VWD1 A, Wavelength=284 nm

RetTime [min]	Sig	Type	Area [mAU*s]	Amt/Area	Amount [ug/mg]	Grp	Name
3.600	1		-	-	-		Pyrogallol
3.942	1	VB	5.66283	1.98812e-2	1.68876		Quinol
4.400	1		-	-	-		Gallic acid

RetTime [min]	Sig	Type	Area [mAU*s]	Amt/Area	Amount [ug/mg]	Grp	Name
7.500	1		-	-	-		Catechol
9.500	1		-	-	-		p- Hydroxy benzoic acid
10.442	1		-	-	-		Caffeine
10.638	1	BV	36.23018	7.29377e-3	3.96382		Chlorgenic
11.171	1	VV	30.26836	9.66124e-3	4.38645		Vanillic acid
11.782	1		-	-	-		Caffeic acid
12.200	1		-	-	-		Syringic acid
13.467	1	VB	14.32416	8.47688e-4	1.82136e-1		Vanillin
15.000	1		-	-	-		p- Coumaric acid
16.400	1		-	-	-		Ferulic acid
17.600	1		-	-	-		Benzoic acid
18.300	1		-	-	-		Rutin
18.600	1		-	-	-		Ellagic
19.115	1	VV	72.06330	2.29527e-3	2.48107		o- Coumaric acid
20.000	1		-	-	-		Salicylic acid
21.358	1	BB	16.40049	1.94922e-1	47.95225		Myricetin
24.500	1		-	-	-		Cinnamic acid
25.040	1	VB	8.41819	2.14640e-2	2.71032		Quercitin
25.800	1		-	-	-		rosemarinic
26.306	1	BV	36.97097	1.10004e-1	61.00421		Neringein
27.426	1	VB	25.24731	6.29345e-2	23.83389		Kampherol

Totals : 148.20290

2 Warnings or Errors :

Warning : Calibration warnings (see calibration table listing)
Warning : Calibrated compound(s) not found

===
*** End of Report ***
