# Supplementary material for: RED light promotes flavonoid and phenolic accumulation in Cichorium spp. callus culture as anti-candida agent
Source: Sci Rep. 2025 Jan 16;15:2194. doi: 10.1038/s41598-024-85099-0 (PMC11739635; doi:10.1038/s41598-024-85099-0)
Supplement: Supplementary file 2 — Supplementary Material 2 [file 41598_2024_85099_MOESM2_ESM.pdf]

===

Acq. Operator : FSQC Lab
Acq. Instrument : Instrument 1 Location : Vial 1
Injection Date : 10/14/2018 2:27:04 PM
Inj Volume : No inj
Acq. Method : C:\CHEM32\1\METHODS\PHENOLS AND FLAVONOIDS2019NEW_LC.M
Last changed : 10/14/2018 2:18:43 PM by FSQC Lab
(modified after loading)
Analysis Method : C:\CHEM32\1\METHODS\PHENOLS AND FLAVONOIDS2019_MIX_1-LOW_LC.M
Last changed : 10/14/2018 3:56:50 PM by FSQC Lab
(modified after loading)
Additional Info : Peak(s) manually integrated

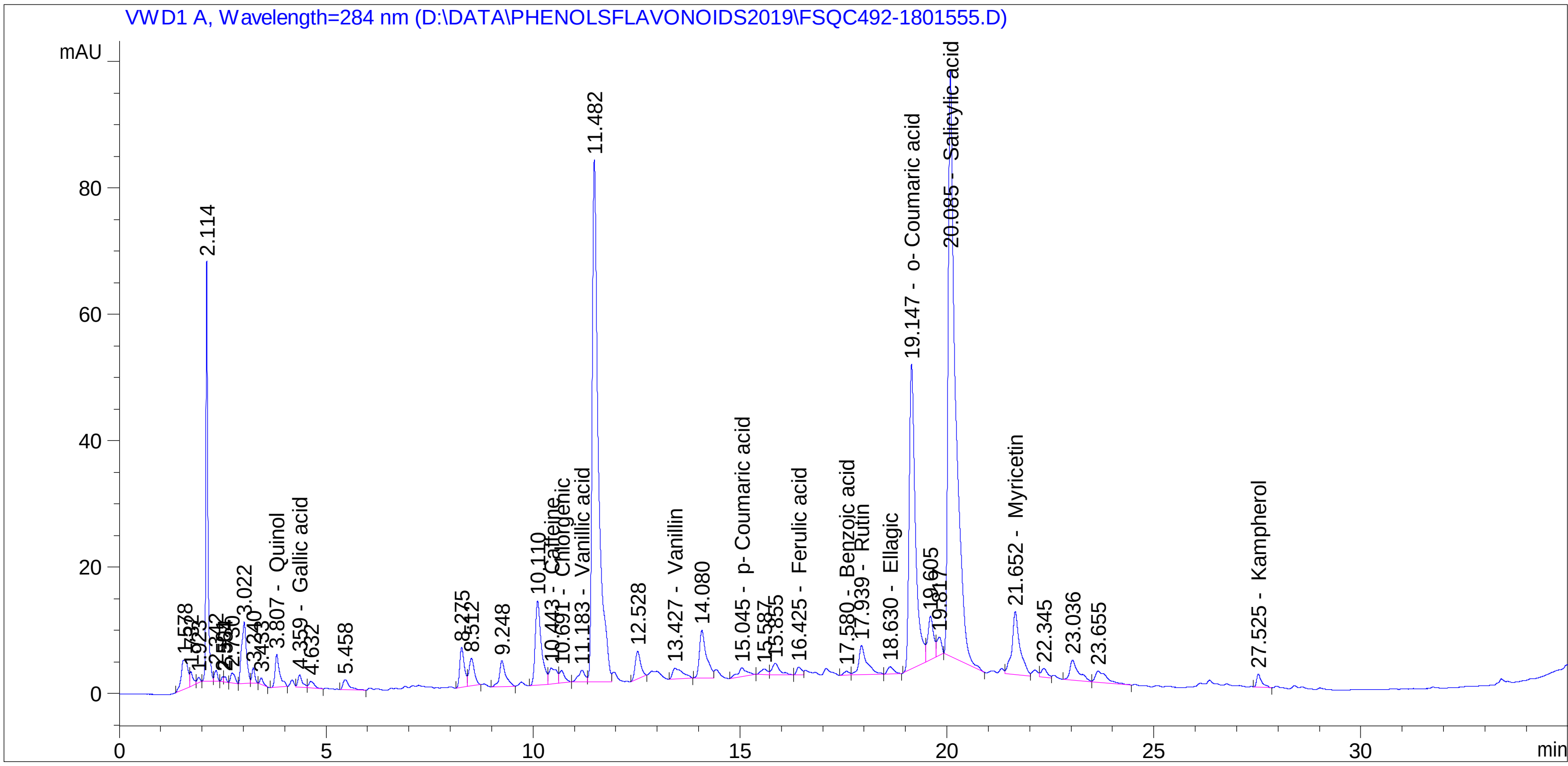

===

External Standard Report

===

Sorted By : Retention Time
Calib. Data Modified : 10/14/2018 3:56:49 PM
Multiplier: : 18.0000
Dilution: : 1.0000
Do not use Multiplier & Dilution Factor with ISTDs

Signal 1: VWD1 A, Wavelength=284 nm

RetTime [min]	Sig	Type	Area [mAU*s]	Amt/Area	Amount [ug/mg]	Grp	Name
3.600	1		-	-	-		Pyrogallol
3.807	1	BB	40.03961	1.99570e-2	14.38325		Quinol
4.359	1	VV	14.65489	7.39610e-3	1.95100		Gallic acid

RetTime [min]	Sig	Type	Area [mAU*s]	Amt/Area	Amount [ug/mg]	Grp	Name
7.500	1		-	-	-		Catechol
9.500	1		-	-	-		p- Hydroxy benzoic acid
10.443	1	VV	32.79903	7.43359e-3	4.38866		Caffeine
10.691	1	VB	17.32305	7.24865e-3	2.26024		Chlorgenic
11.183	1	BV	21.06964	8.08350e-3	3.06570		Vanillic acid
11.782	1		-	-	-		Caffeic acid
12.200	1		-	-	-		Syringic acid
13.427	1	BB	27.64201	3.51143e-3	1.74713		Vanillin
15.045	1	BB	21.18777	2.90032e-4	1.10613e-1		p- Coumaric acid
16.425	1	BV	11.15702	0.00000	0.00000		Ferulic acid
17.580	1	BV	5.75982	8.51337e-2	8.82639		Benzoic acid
17.939	1	VB	62.42522	3.18071e-2	35.74016		Rutin
18.630	1	BB	11.82248	1.11690e-1	23.76823		Ellagic
19.147	1	BV	502.63547	4.17509e-3	37.77391		o- Coumaric acid
20.085	1	BB	1150.62671	3.15493e-2	653.42711		Salicylic acid
21.652	1	VV	133.36652	1.15586e-1	277.47572		Myricetin
24.500	1		-	-	-		Cinnamic acid
25.200	1		-	-	-		Quercitin
25.800	1		-	-	-		rosemarinic
26.500	1		-	-	-		Neringein
27.525	1	BB	17.85291	6.23333e-2	20.03094		Kampherol

Totals : 1084.94904

3 Warnings or Errors :

Warning : Calibration warnings (see calibration table listing)
Warning : Calibrated compound(s) not found
Warning : Negative results set to zero (cal. curve intercept), (Ferulic acid)

==

*** End of Report ***
