# Supplementary material for: RED light promotes flavonoid and phenolic accumulation in Cichorium spp. callus culture as anti-candida agent
Source: Sci Rep. 2025 Jan 16;15:2194. doi: 10.1038/s41598-024-85099-0 (PMC11739635; doi:10.1038/s41598-024-85099-0)
Supplement: Supplementary file 3 — Supplementary Material 3 [file 41598_2024_85099_MOESM3_ESM.pdf]

===

Acq. Operator : FSQC Lab
Acq. Instrument : Instrument 1 Location : Vial 1
Injection Date : 10/30/2018 4:05:22 PM
Inj Volume : No inj
Acq. Method : C:\CHEM32\1\METHODS\PHENOLS AND FLAVONOIDS2019NEW_LC.M
Last changed : 10/30/2018 3:54:25 PM by FSQC Lab
Analysis Method : C:\CHEM32\1\METHODS\PHENOLS AND FLAVONOIDS2019_MIX_1_LC.M
Last changed : 11/15/2018 11:05:14 AM by FSQC Lab
(modified after loading)
Additional Info : Peak(s) manually integrated

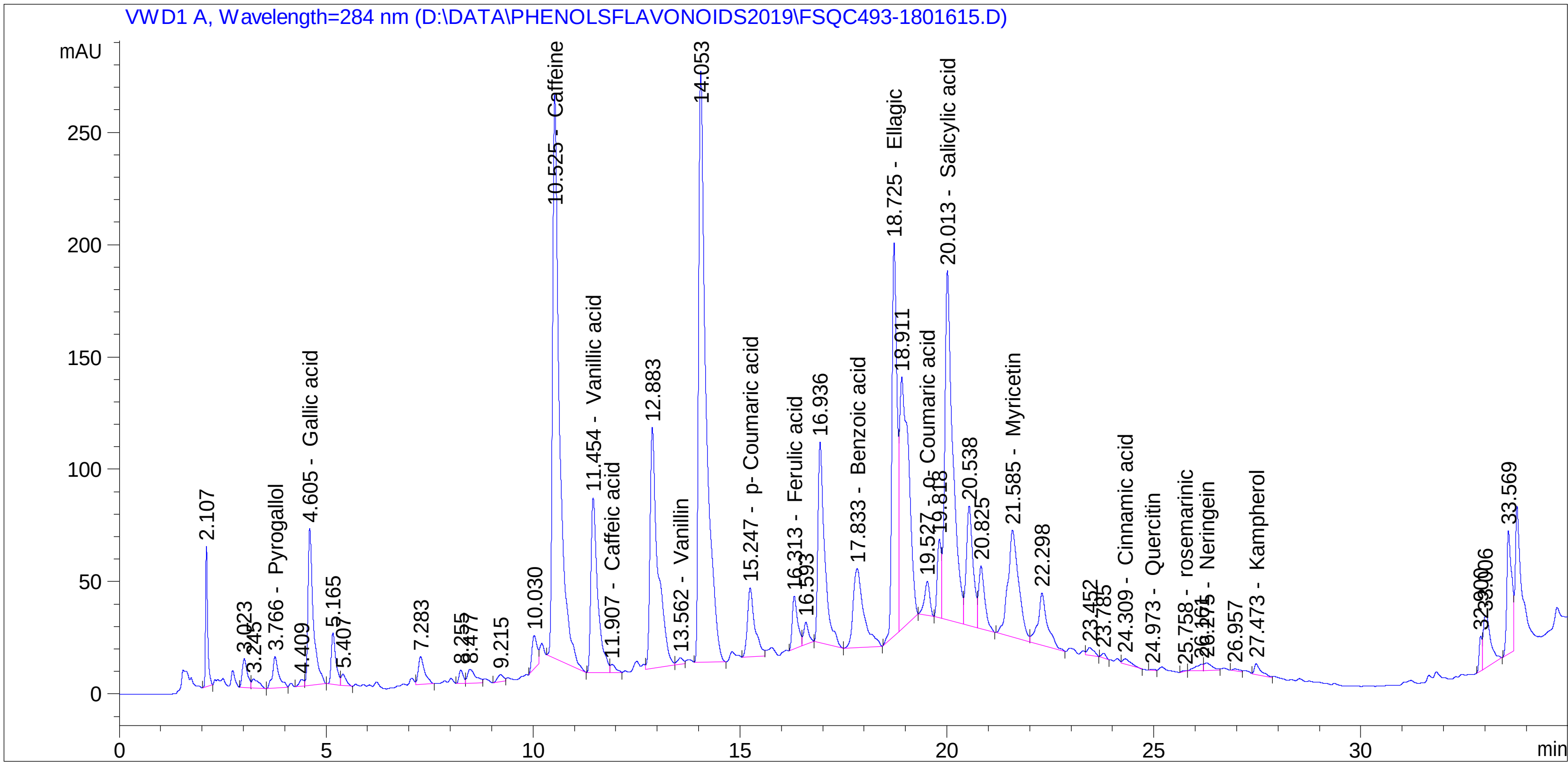

===

External Standard Report

===

Sorted By : Retention Time
Calib. Data Modified : 11/7/2018 11:56:56 AM
Multiplier: : 18.0000
Dilution: : 1.0000
Do not use Multiplier & Dilution Factor with ISTDs

Signal 1: VWD1 A, Wavelength=284 nm

RetTime [min]	Sig	Type	Area [mAU*s]	Amt/Area	Amount [ppm]	Grp	Name
3.766	1	BV	152.05663	1.12263e-2	30.72654		Pyrogallol
4.100	1		-	-	-		Quinol
4.605	1	VB	631.93506	9.35061e-3	106.36156		Gallic acid
7.500	1		-	-	-		Catechol

RetTime [min]	Sig	Type	Area [mAU*s]	Amt/Area	Amount [ppm]	Grp	Name
9.500	1		-	-	-		p- Hydroxy benzoic acid
10.525	1	BB	2921.95215	1.21841e-2	640.82597		Caffeine
10.800	1		-	-	-		Chlorgenic
11.454	1	BV	889.34497	1.31521e-2	210.54098		Vanillic acid
11.907	1	VB	32.62342	2.82492e-3	1.65885		Caffeic acid
12.200	1		-	-	-		Syringic acid
13.562	1	VV	28.11120	3.55925e-3	1.80099		Vanillin
15.247	1	BV	380.28073	4.16605e-3	28.51684		p- Coumaric acid
16.313	1	BV	211.62564	6.85811e-3	26.12433		Ferulic acid
17.833	1	BB	624.25989	9.90037e-2	1112.47238		Benzoic acid
18.300	1		-	-	-		Rutin
18.725	1	BV	1480.98474	2.51436e-1	6702.70697		Ellagic
19.527	1	BB	134.69478	3.42711e-3	8.30906		o- Coumaric acid
20.013	1	VV	2105.13135	3.16776e-2	1200.33839		Salicylic acid
21.585	1	BV	862.22906	1.06183e-1	1647.96716		Myricetin
24.309	1	VB	33.27100	1.95088e-4	1.16834e-1		Cinnamic acid
24.973	1	BV	4.11076	0.00000	0.00000		Quercitin
25.758	1	BB	2.61405	1.97991e-1	9.31604		rosemarinic
26.275	1	VV	45.88200	1.02286e-1	84.47569		Neringein
27.473	1	VV	53.56577	6.37018e-2	61.42027		Kampherol

Totals : 1.18737e4

3 Warnings or Errors :

Warning : Calibration warnings (see calibration table listing)
Warning : Calibrated compound(s) not found
Warning : Negative results set to zero (cal. curve intercept), (Quercitin)

==
*** End of Report ***
