# Supplementary material for: RED light promotes flavonoid and phenolic accumulation in Cichorium spp. callus culture as anti-candida agent
Source: Sci Rep. 2025 Jan 16;15:2194. doi: 10.1038/s41598-024-85099-0 (PMC11739635; doi:10.1038/s41598-024-85099-0)
Supplement: Supplementary file 4 — Supplementary Material 4 [file 41598_2024_85099_MOESM4_ESM.pdf]

Data File D:\DATA\PHENOLSFLAVONOIDS2019\FSQC494-1801559.D
Sample Name: FSQC494-18

===

Acq. Operator : FSQC Lab
Acq. Instrument : Instrument 1 Location : Vial 1
Injection Date : 10/15/2018 1:59:20 PM
Inj Volume : No inj
Acq. Method : C:\CHEM32\1\METHODS\PHENOLS AND FLAVONOIDS2019_MIX_1-LOW_LC.M
Last changed : 10/15/2018 1:19:37 PM by FSQC Lab
(modified after loading)
Analysis Method : C:\CHEM32\1\METHODS\PHENOLS AND FLAVONOIDS2019_MIX_1-LOW_LC.M
Last changed : 10/15/2018 2:42:51 PM by FSQC Lab
(modified after loading)
Additional Info : Peak(s) manually integrated

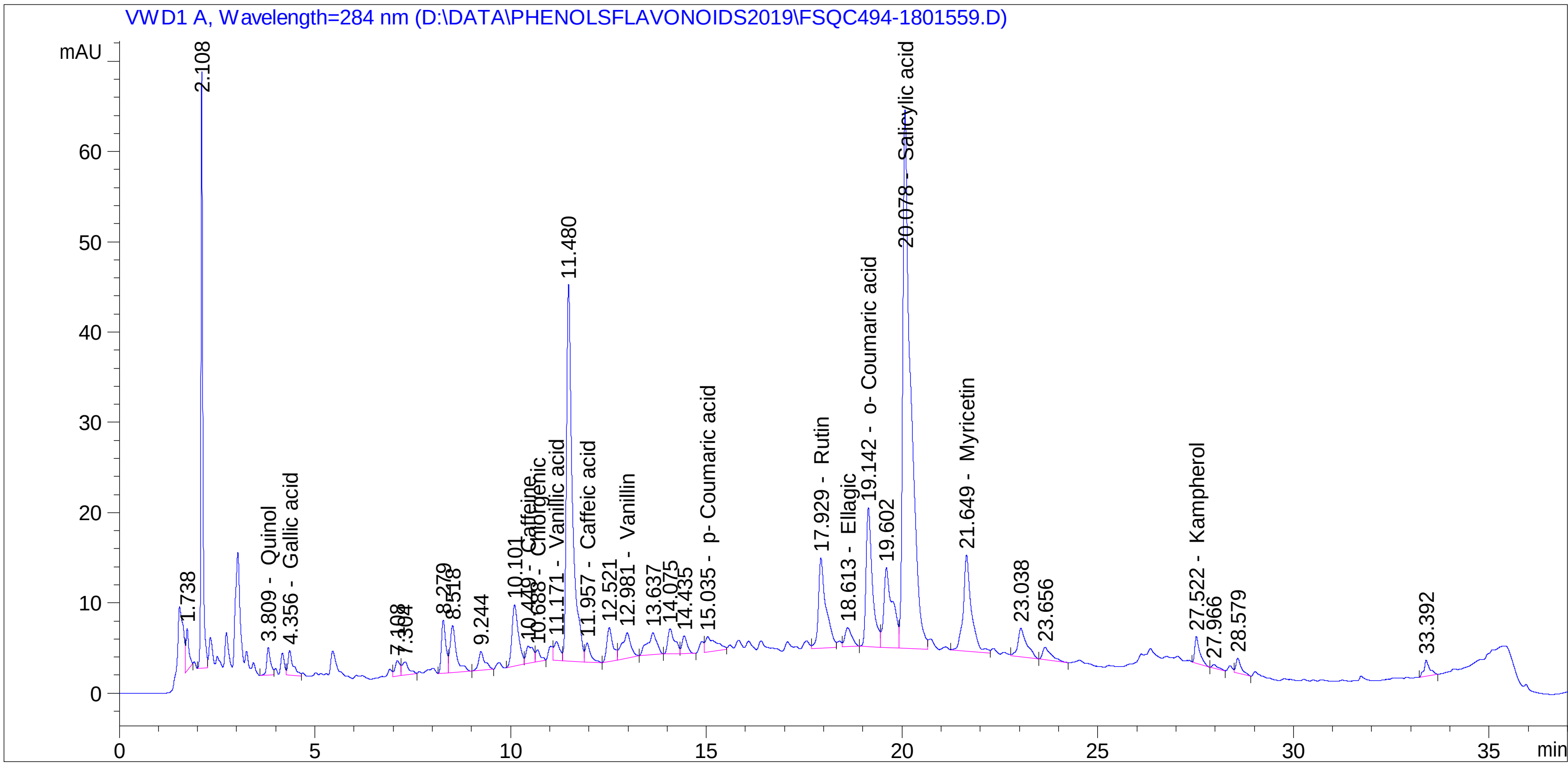

===

External Standard Report

===

Sorted By : Retention Time
Calib. Data Modified : 10/15/2018 1:57:56 PM
Multiplier: : 18.0000
Dilution: : 1.0000
Do not use Multiplier & Dilution Factor with ISTDs

Signal 1: VWD1 A, Wavelength=284 nm

RetTime [min]	Sig	Type	Area [mAU*s]	Amt/Area	Amount [ug/mg]	Grp	Name
3.600	1		-	-	-		Pyrogallol
3.809	1	BV	21.19790	1.99459e-2	7.61060		Quinol
4.356	1	VV	24.96987	6.80496e-3	3.05854		Gallic acid

RetTime [min]	Sig	Type	Area [mAU*s]	Amt/Area	Amount [ug/mg]	Grp	Name
7.500	1		-	-	-		Catechol
9.500	1		-	-	-		p- Hydroxy benzoic acid
10.449	1	VV	24.44273	5.79108e-3	2.54790		Caffeine
10.688	1	VB	10.75661	7.19587e-3	1.39326		Chlorgenic
11.171	1	VV	21.55482	8.20036e-3	3.18163		Vanillic acid
11.957	1	VB	20.34994	6.21083e-4	2.27502e-1		Caffeic acid
12.200	1		-	-	-		Syringic acid
12.981	1	VB	44.32663	4.58983e-3	3.66213		Vanillin
15.035	1	VV	31.05509	1.59425e-3	8.91172e-1		p- Coumaric acid
16.400	1		-	-	-		Ferulic acid
17.600	1		-	-	-		Benzoic acid
17.929	1	VV	132.49457	3.62804e-2	86.52519		Rutin
18.613	1	VB	26.34458	1.89343e-1	89.78695		Ellagic
19.142	1	BV	173.63251	3.77703e-3	11.80468		o- Coumaric acid
20.078	1	VV	846.44922	3.14477e-2	479.13974		Salicylic acid
21.649	1	BV	152.53612	1.14188e-1	313.52061		Myricetin
24.500	1		-	-	-		Cinnamic acid
25.200	1		-	-	-		Quercitin
25.800	1		-	-	-		rosemarinic
26.500	1		-	-	-		Neringein
27.522	1	BB	25.87010	6.29694e-2	29.32244		Kampherol

Totals : 1032.67234

2 Warnings or Errors :

Warning : Calibration warnings (see calibration table listing)
Warning : Calibrated compound(s) not found

===

*** End of Report ***
