# Supplementary material for: RED light promotes flavonoid and phenolic accumulation in Cichorium spp. callus culture as anti-candida agent
Source: Sci Rep. 2025 Jan 16;15:2194. doi: 10.1038/s41598-024-85099-0 (PMC11739635; doi:10.1038/s41598-024-85099-0)
Supplement: Supplementary file 5 — Supplementary Material 5 [file 41598_2024_85099_MOESM5_ESM.pdf]

===

Acq. Operator : FSQC Lab
Acq. Instrument : Instrument 1 Location : Vial 1
Injection Date : 10/17/2018 9:50:28 AM
Inj Volume : No inj
Acq. Method : C:\CHEM32\1\METHODS\PHENOLS AND FLAVONOIDS2019_MIX_1-LOW_LC.M
Last changed : 10/17/2018 9:50:27 AM by FSQC Lab
(modified after loading)
Analysis Method : C:\CHEM32\1\METHODS\PHENOLS AND FLAVONOIDS2019_MIX_1-LOW_LC.M
Last changed : 10/17/2018 1:07:49 PM by FSQC Lab
(modified after loading)
Additional Info : Peak(s) manually integrated

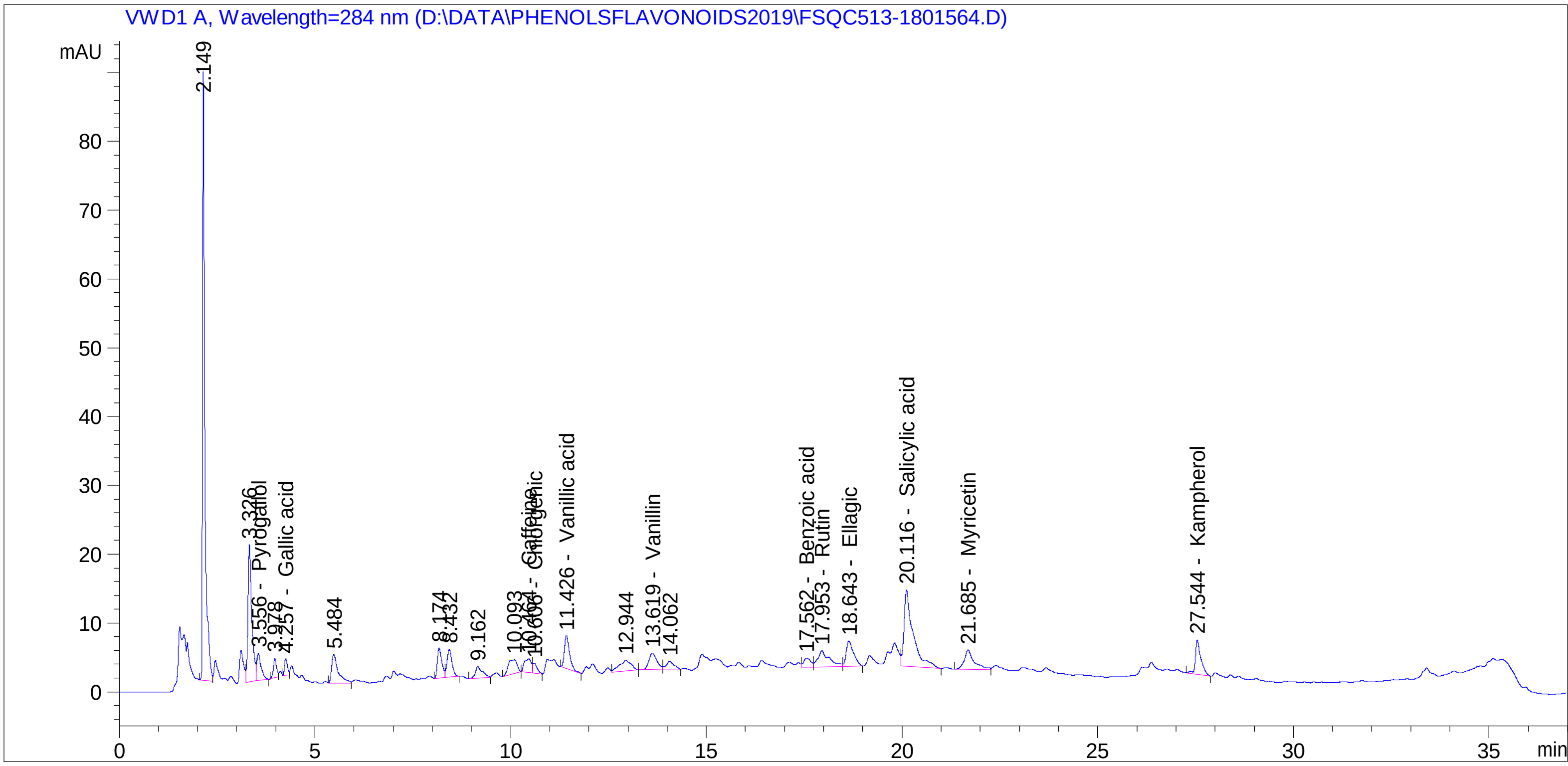

===

External Standard Report

===

Sorted By : Retention Time
Calib. Data Modified : 10/16/2018 4:23:26 PM
Multiplier: : 19.0000
Dilution: : 1.0000
Do not use Multiplier & Dilution Factor with ISTDs

Signal 1: VWD1 A, Wavelength=284 nm

RetTime [min]	Sig	Type	Area [mAU*s]	Amt/Area	Amount [ug/mg]	Grp	Name
3.556	1	VB	30.41304	1.13094e-2	6.53509		Pyrogallol
3.800	1		-	-	-		Quinol
4.257	1	BV	13.39297	7.53093e-3	1.91637		Gallic acid

RetTime [min]	Sig	Type	Area [mAU*s]	Amt/Area	Amount [ug/mg]	Grp	Name
7.500	1		-	-	-		Catechol
9.500	1		-	-	-		p- Hydroxy benzoic acid
10.464	1	BV	24.64797	5.84476e-3	2.73717		Caffeine
10.606	1	VB	11.04761	7.19953e-3	1.51122		Chlorgenic
11.426	1	BB	43.57089	1.07646e-2	8.91141		Vanillic acid
11.782	1		-	-	-		Caffeic acid
12.200	1		-	-	-		Syringic acid
13.619	1	BV	34.39611	4.07401e-3	2.66247		Vanillin
15.000	1		-	-	-		p- Coumaric acid
16.400	1		-	-	-		Ferulic acid
17.562	1	VV	16.45098	9.42315e-2	29.45381		Benzoic acid
17.953	1	VV	47.14010	2.90644e-2	26.03186		Rutin
18.643	1	VB	47.11011	2.17208e-1	194.42154		Ellagic
19.300	1		-	-	-		o- Coumaric acid
20.116	1	VB	177.93074	3.00031e-2	101.43105		Salicylic acid
21.685	1	BV	50.51362	1.33832e-1	128.44648		Myricetin
24.500	1		-	-	-		Cinnamic acid
25.200	1		-	-	-		Quercitin
25.800	1		-	-	-		rosemarinic
26.500	1		-	-	-		Neringein
27.544	1	BB	48.60850	6.36321e-2	58.76812		Kampherol

Totals : 562.82660

2 Warnings or Errors :

Warning : Calibration warnings (see calibration table listing)
Warning : Calibrated compound(s) not found

==
*** End of Report ***
