# Supplementary material for: RED light promotes flavonoid and phenolic accumulation in Cichorium spp. callus culture as anti-candida agent
Source: Sci Rep. 2025 Jan 16;15:2194. doi: 10.1038/s41598-024-85099-0 (PMC11739635; doi:10.1038/s41598-024-85099-0)
Supplement: Supplementary file 6 — Supplementary Material 6 [file 41598_2024_85099_MOESM6_ESM.pdf]

Data File D:\DATA\PHENOLSFLAVONOIDS2019\FSQC512-1801558.D
Sample Name: FSQC512-18

```
=====================================================================
Acq. Operator   : FSQC Lab
Acq. Instrument : Instrument 1                    Location : Vial 1
Injection Date  : 10/15/2018 12:42:34 PM
                                                Inj Volume : No inj
Acq. Method     : C:\CHEM32\1\METHODS\PHENOLS AND FLAVONOIDS2019_MIX_1-LOW_LC.M
Last changed    : 10/15/2018 12:28:31 PM by FSQC Lab
                  (modified after loading)
Analysis Method : C:\CHEM32\1\METHODS\PHENOLS AND FLAVONOIDS2019_MIX_1-LOW_LC.M
Last changed    : 10/15/2018 1:57:57 PM by FSQC Lab
                  (modified after loading)
Additional Info : Peak(s) manually integrated
```

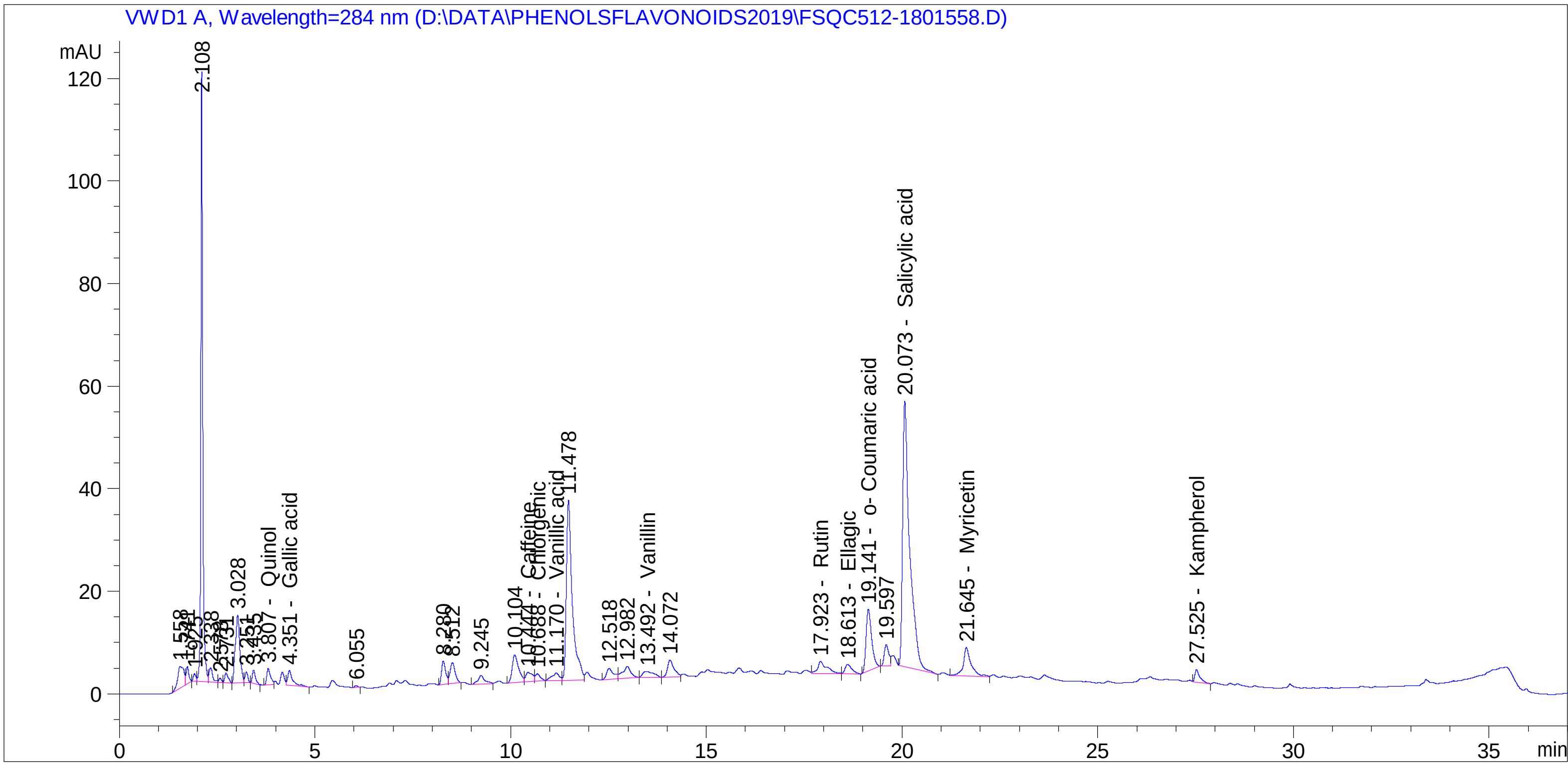


```
=====================================================================
                     External Standard Report
=====================================================================

Sorted By             :      Retention Time
Calib. Data Modified  :      10/15/2018 1:57:56 PM
Multiplier:                    :     19.0000
Dilution:                      :      1.0000
Do not use Multiplier & Dilution Factor with ISTDs

Signal 1: VWD1 A, Wavelength=284 nm
```

RetTime [min]	Sig	Type	Area [mAU*s]	Amt/Area	Amount [ug/mg]	Grp	Name
3.600	1		-	-	-		Pyrogallol
3.807	1	BV	22.36916	1.99471e-2	8.47781		Quinol
4.351	1	VB	26.40938	6.75918e-3	3.39161		Gallic acid

RetTime [min]	Sig	Type	Area [mAU*s]	Amt/Area	Amount [ug/mg]	Grp	Name
7.500	1		-	-	-		Catechol
9.500	1		-	-	-		p- Hydroxy benzoic acid
10.444	1	VV	22.93984	5.36871e-3	2.33999		Caffeine
10.688	1	VB	13.01896	7.22006e-3	1.78596		Chlorgenic
11.170	1	BV	19.46834	7.65649e-3	2.83212		Vanillic acid
11.782	1		-	-	-		Caffeic acid
12.200	1		-	-	-		Syringic acid
13.492	1	BB	21.78451	2.74107e-3	1.13455		Vanillin
15.000	1		-	-	-		p- Coumaric acid
16.400	1		-	-	-		Ferulic acid
17.600	1		-	-	-		Benzoic acid
17.923	1	VB	38.29209	2.64761e-2	19.26271		Rutin
18.613	1	BB	21.01160	1.73298e-1	69.18400		Ellagic
19.141	1	BB	120.50761	3.50894e-3	8.03422		o- Coumaric acid
20.073	1	BB	648.07556	3.13300e-2	385.77998		Salicylic acid
21.645	1	BV	77.61967	1.23576e-1	182.24595		Myricetin
24.500	1		-	-	-		Cinnamic acid
25.200	1		-	-	-		Quercitin
25.800	1		-	-	-		rosemarinic
26.500	1		-	-	-		Neringein
27.525	1	VB	22.61292	6.27654e-2	26.96685		Kampherol

Totals : 711.43575

2 Warnings or Errors :

Warning : Calibration warnings (see calibration table listing)
Warning : Calibrated compound(s) not found

==
*** End of Report ***
