# Supplementary material for: RED light promotes flavonoid and phenolic accumulation in Cichorium spp. callus culture as anti-candida agent
Source: Sci Rep. 2025 Jan 16;15:2194. doi: 10.1038/s41598-024-85099-0 (PMC11739635; doi:10.1038/s41598-024-85099-0)
Supplement: Supplementary file 7 — Supplementary Material 7 [file 41598_2024_85099_MOESM7_ESM.pdf]

Data File D:\DATA\PHENOLSFLAVONOIDS2019\FSQC519-1801623.D
Sample Name: FSQC519-18

===

Acq. Operator : FSQC Lab
Acq. Instrument : Instrument 1 Location : Vial 1
Injection Date : 10/31/2018 3:20:56 PM
Inj Volume : No inj
Acq. Method : C:\CHEM32\1\METHODS\PHENOLS AND FLAVONOIDS2019NEW_LC.M
Last changed : 10/31/2018 3:03:05 PM by FSQC Lab
(modified after loading)
Analysis Method : C:\CHEM32\1\METHODS\PHENOLS AND FLAVONOIDS2019_MIX_1_LC.M
Last changed : 11/25/2018 1:45:30 PM by FSQC Lab
(modified after loading)
Additional Info : Peak(s) manually integrated

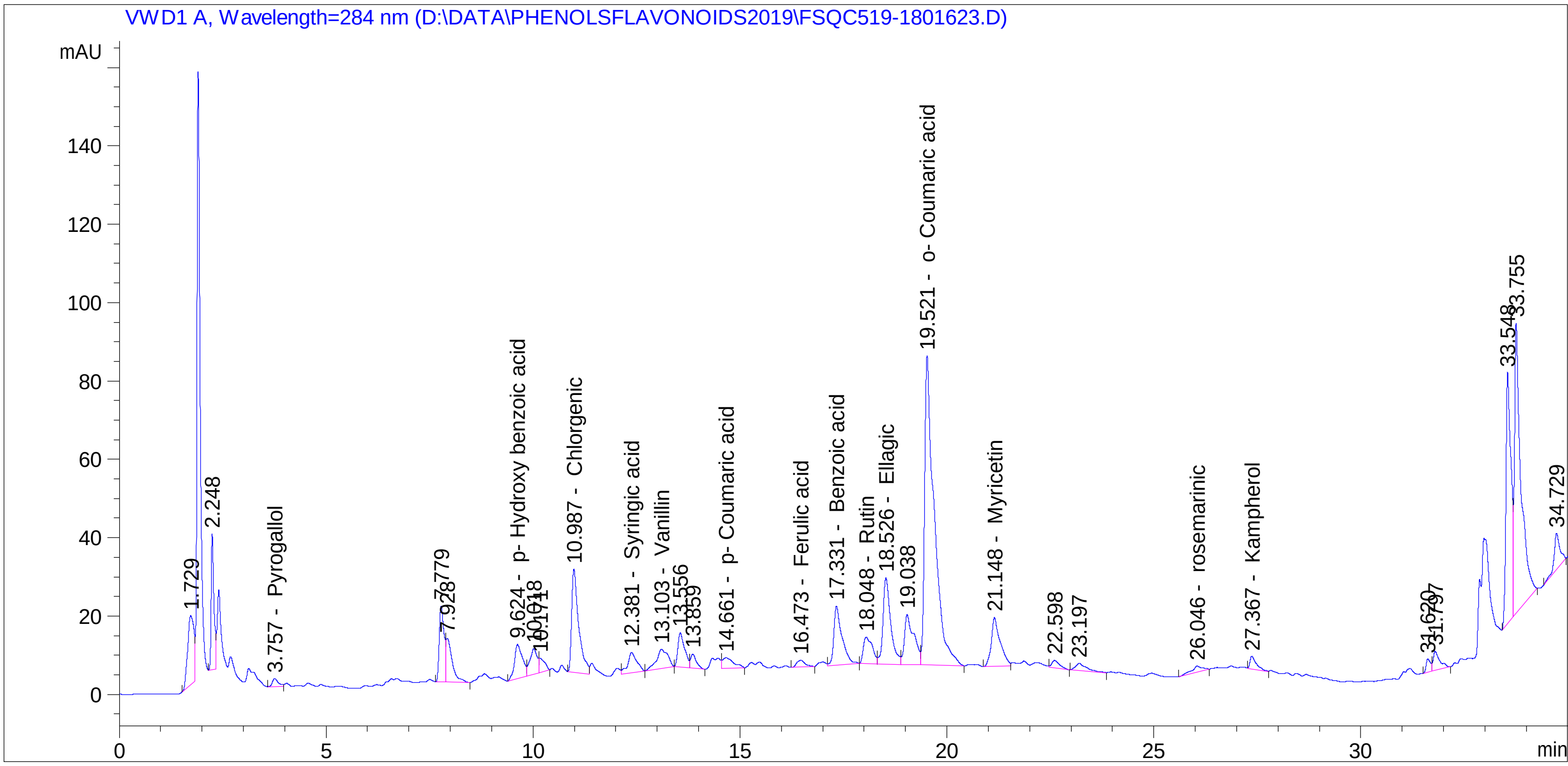

===

External Standard Report

===

Sorted By : Retention Time
Calib. Data Modified : 11/25/2018 1:29:57 PM
Multiplier: : 21.0000
Dilution: : 1.0000
Do not use Multiplier & Dilution Factor with ISTDs

Signal 1: VWD1 A, Wavelength=284 nm

RetTime [min]	Sig	Type	Area [mAU*s]	Amt/Area	Amount [ppm]	Grp	Name
3.757	1	BV	20.85167	1.13570e-2	4.97306		Pyrogallol
3.900	1		-	-	-		Quinol
4.120	1		-	-	-		Gallic acid

RetTime [min]	Sig	Type	Area [mAU*s]	Amt/Area	Amount [ppm]	Grp	Name
7.500	1		-	-	-		Catechol
9.624	1	BV	116.10089	2.69764e-2	65.77164		p- Hydroxy benzoic acid
10.442	1		-	-	-		Caffeine
10.987	1	BV	309.30170	7.33027e-3	47.61256		Chlorgenic
11.350	1		-	-	-		Vanillic acid
11.782	1		-	-	-		Caffeic acid
12.381	1	VB	80.38803	7.57090e-3	12.78080		Syringic acid
13.103	1	BB	92.00638	5.51569e-3	10.65706		Vanillin
14.661	1	VB	50.58093	2.67533e-3	2.84174		p- Coumaric acid
16.473	1	VB	24.59638	2.07106e-3	1.06975		Ferulic acid
17.331	1	VB	205.01350	9.87395e-2	425.10169		Benzoic acid
18.048	1	BV	103.37132	3.51576e-2	76.32004		Rutin
18.526	1	VV	286.94446	2.46756e-1	1486.91263		Ellagic
19.521	1	VB	1285.96179	4.59300e-3	124.03477		o- Coumaric acid
20.000	1		-	-	-		Salicylic acid
21.148	1	BV	185.18466	1.12473e-1	437.39520		Myricetin
24.500	1		-	-	-		Cinnamic acid
25.200	1		-	-	-		Quercitin
26.046	1	BB	26.77363	1.14940e-1	64.62458		rosemarinic
26.500	1		-	-	-		Neringein
27.367	1	VV	38.18274	6.34262e-2	50.85751		Kampherol

Totals : 2810.95303

2 Warnings or Errors :

Warning : Calibration warnings (see calibration table listing)
Warning : Calibrated compound(s) not found

===
*** End of Report ***
