# Supplementary material for: RED light promotes flavonoid and phenolic accumulation in Cichorium spp. callus culture as anti-candida agent
Source: Sci Rep. 2025 Jan 16;15:2194. doi: 10.1038/s41598-024-85099-0 (PMC11739635; doi:10.1038/s41598-024-85099-0)
Supplement: Supplementary file 8 — Supplementary Material 8 [file 41598_2024_85099_MOESM8_ESM.pdf]

Data File D:\DATA\PHENOLSFLAVONOIDS2019\FSQC520-1801611.D
Sample Name: FSQC520-18

===

Acq. Operator : FSQC Lab
Acq. Instrument : Instrument 1 Location : Vial 1
Injection Date : 10/30/2018 1:08:21 PM
Inj Volume : No inj
Acq. Method : C:\CHEM32\1\METHODS\PHENOLS AND FLAVONOIDS2019NEW_LC.M
Last changed : 10/30/2018 12:40:51 PM by FSQC Lab
Analysis Method : C:\CHEM32\1\METHODS\PHENOLS AND FLAVONOIDS2019_MIX_1_LC.M
Last changed : 11/25/2018 1:58:15 PM by FSQC Lab
(modified after loading)
Additional Info : Peak(s) manually integrated

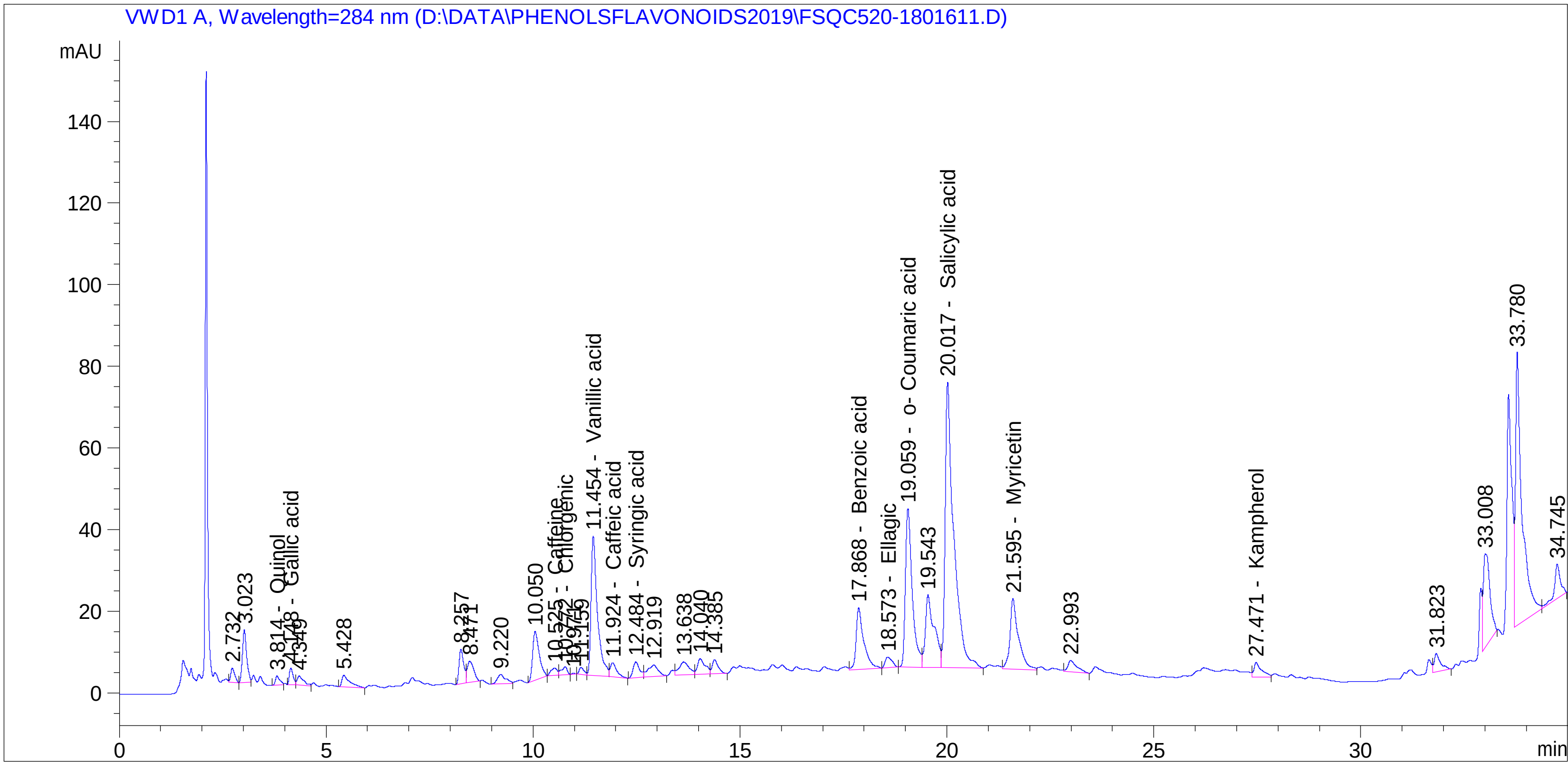

===
External Standard Report
===

Sorted By : Retention Time
Calib. Data Modified : 11/25/2018 1:29:57 PM
Multiplier: : 20.0000
Dilution: : 1.0000
Do not use Multiplier & Dilution Factor with ISTDs

Signal 1: VWD1 A, Wavelength=284 nm

RetTime [min]	Sig	Type	Area [mAU*s]	Amt/Area	Amount [ppm]	Grp	Name
3.720	1		-	-	-		Pyrogallol
3.814	1	BV	16.90859	1.99399e-2	6.74311		Quinol
4.148	1	BV	26.43680	3.42427e-3	1.81054		Gallic acid
7.500	1		-	-	-		Catechol

RetTime [min]	Sig	Type	Area [mAU*s]	Amt/Area	Amount [ppm]	Grp	Name
9.500	1		-	-	-		p- Hydroxy benzoic acid
10.525	1	BV	18.30675	3.63021e-3	1.32915		Caffeine
10.772	1	VB	19.26055	7.25735e-3	2.79561		Chlorgenic
11.454	1	VV	367.17191	1.29771e-2	95.29688		Vanillic acid
11.924	1	VB	38.68655	3.39759e-3	2.62882		Caffeic acid
12.484	1	BV	42.21555	7.05835e-3	5.95944		Syringic acid
13.300	1		-	-	-		Vanillin
15.000	1		-	-	-		p- Coumaric acid
16.400	1		-	-	-		Ferulic acid
17.868	1	VB	193.27341	9.87156e-2	381.58217		Benzoic acid
18.300	1		-	-	-		Rutin
18.573	1	BB	30.34543	1.97678e-1	119.97238		Ellagic
19.059	1	BV	448.39301	4.33820e-3	38.90438		o- Coumaric acid
20.017	1	VB	1015.53333	3.15117e-2	640.02379		Salicylic acid
21.595	1	VV	247.05383	1.10467e-1	545.82636		Myricetin
24.500	1		-	-	-		Cinnamic acid
25.200	1		-	-	-		Quercitin
25.800	1		-	-	-		rosemarinic
26.500	1		-	-	-		Neringein
27.471	1	VV	45.74275	6.35848e-2	58.17090		Kampherol

Totals : 1901.04352

2 Warnings or Errors :

Warning : Calibration warnings (see calibration table listing)
Warning : Calibrated compound(s) not found

===
*** End of Report ***
