# Supplementary material for: RED light promotes flavonoid and phenolic accumulation in Cichorium spp. callus culture as anti-candida agent
Source: Sci Rep. 2025 Jan 16;15:2194. doi: 10.1038/s41598-024-85099-0 (PMC11739635; doi:10.1038/s41598-024-85099-0)
Supplement: Supplementary file 9 — Supplementary Material 9 [file 41598_2024_85099_MOESM9_ESM.pdf]

Data File D:\DATA\PHENOLSFLAVONOIDS2019\FSQC518-1801621.D
Sample Name: FSQC518-18

===

Acq. Operator : FSQC Lab
Acq. Instrument : Instrument 1 Location : Vial 1
Injection Date : 10/31/2018 1:13:27 PM
Inj Volume : No inj
Acq. Method : C:\CHEM32\1\METHODS\PHENOLS AND FLAVONOIDS2019NEW_LC.M
Last changed : 10/31/2018 12:46:46 PM by FSQC Lab
(modified after loading)
Analysis Method : C:\CHEM32\1\METHODS\PHENOLS AND FLAVONOIDS2019_MIX_1_LC.M
Last changed : 11/25/2018 1:45:30 PM by FSQC Lab
(modified after loading)
Additional Info : Peak(s) manually integrated

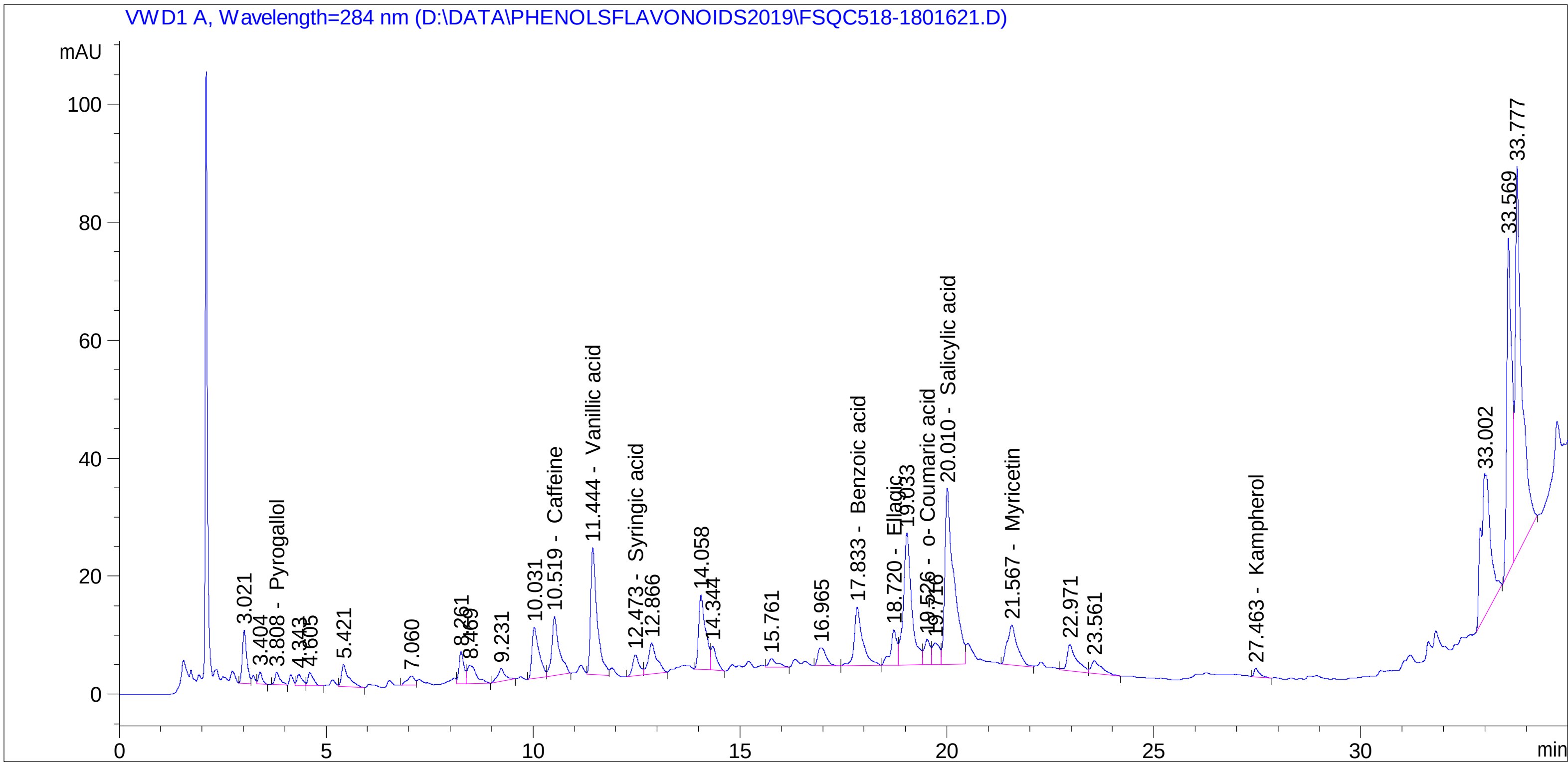

===

External Standard Report

===

Sorted By : Retention Time
Calib. Data Modified : 11/25/2018 1:29:57 PM
Multiplier: : 21.0000
Dilution: : 1.0000
Do not use Multiplier & Dilution Factor with ISTDs

Signal 1: VWD1 A, Wavelength=284 nm

RetTime [min]	Sig	Type	Area [mAU*s]	Amt/Area	Amount [ppm]	Grp	Name
3.808	1	BB	18.16058	1.13794e-2	4.33981		Pyrogallol
3.900	1		-	-	-		Quinol
4.120	1		-	-	-		Gallic acid

RetTime [min]	Sig	Type	Area [mAU*s]	Amt/Area	Amount [ppm]	Grp	Name
7.500	1		-	-	-		Catechol
9.500	1		-	-	-		p- Hydroxy benzoic acid
10.519	1	VB	124.96059	1.09770e-2	28.80556		Caffeine
10.800	1		-	-	-		Chlorgenic
11.444	1	VV	235.40813	1.28104e-2	63.32911		Vanillic acid
11.782	1		-	-	-		Caffeic acid
12.473	1	BV	39.57841	6.98643e-3	5.80674		Syringic acid
13.300	1		-	-	-		Vanillin
15.000	1		-	-	-		p- Coumaric acid
16.400	1		-	-	-		Ferulic acid
17.833	1	BB	140.38271	9.85585e-2	290.55397		Benzoic acid
18.300	1		-	-	-		Rutin
18.720	1	BV	65.17162	2.27006e-1	310.68101		Ellagic
19.526	1	VV	44.53026	7.90252e-4	7.38993e-1		o- Coumaric acid
20.010	1	VV	458.52789	3.11224e-2	299.68038		Salicylic acid
21.567	1	BV	114.61208	1.17406e-1	282.58005		Myricetin
24.500	1		-	-	-		Cinnamic acid
25.200	1		-	-	-		Quercitin
25.800	1		-	-	-		rosemarinic
26.500	1		-	-	-		Neringein
27.463	1	BB	13.89183	6.17480e-2	18.01364		Kampherol

Totals : 1304.52926

2 Warnings or Errors :

Warning : Calibration warnings (see calibration table listing)
Warning : Calibrated compound(s) not found

==

*** End of Report ***
