# Supplementary material for: RED light promotes flavonoid and phenolic accumulation in Cichorium spp. callus culture as anti-candida agent
Source: Sci Rep. 2025 Jan 16;15:2194. doi: 10.1038/s41598-024-85099-0 (PMC11739635; doi:10.1038/s41598-024-85099-0)
Supplement: Supplementary file 10 — Supplementary Material 10 [file 41598_2024_85099_MOESM10_ESM.pdf]

Data File D:\DATA\PHENOLSFLAVONOIDS2019\FSQC514-1801612.D
Sample Name: FSQC514-18

===

Acq. Operator : FSQC Lab
Acq. Instrument : Instrument 1 Location : Vial 1
Injection Date : 10/30/2018 1:46:34 PM
Inj Volume : No inj
Acq. Method : C:\CHEM32\1\METHODS\PHENOLS AND FLAVONOIDS2019NEW_LC.M
Last changed : 10/30/2018 1:43:25 PM by FSQC Lab
Analysis Method : C:\CHEM32\1\METHODS\PHENOLS AND FLAVONOIDS2019_MIX_1_LC.M
Last changed : 11/25/2018 1:02:06 PM by FSQC Lab
(modified after loading)
Additional Info : Peak(s) manually integrated

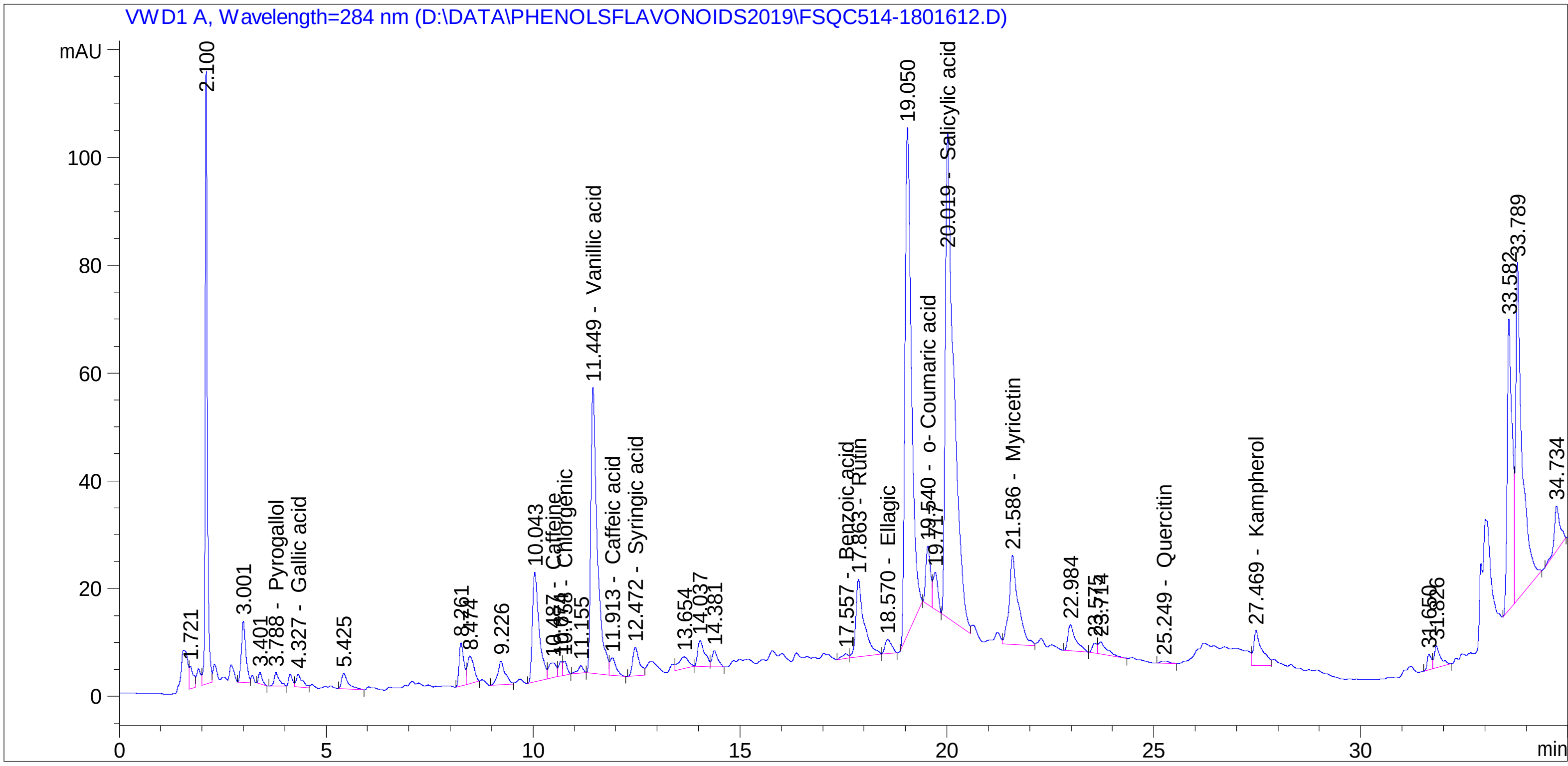

===

External Standard Report

===

Sorted By : Retention Time
Calib. Data Modified : 11/25/2018 12:59:53 PM
Multiplier: : 19.0000
Dilution: : 1.0000
Do not use Multiplier & Dilution Factor with ISTDs

Signal 1: VWD1 A, Wavelength=284 nm

RetTime [min]	Sig	Type	Area [mAU*s]	Amt/Area	Amount [ppm]	Grp	Name
3.788	1	BB	21.56936	1.13520e-2	4.65223		Pyrogallol
4.000	1		-	-	-		Quinol
4.327	1	VV	24.39509	2.90662e-3	1.34724		Gallic acid
7.500	1		-	-	-		Catechol

RetTime [min]	Sig	Type	Area [mAU*s]	Amt/Area	Amount [ppm]	Grp	Name
9.500	1		-	-	-		p- Hydroxy benzoic acid
10.487	1	VV	35.78736	7.83478e-3	5.32733		Caffeine
10.758	1	VB	18.26158	7.25309e-3	2.51661		Chlorgenic
11.449	1	BV	579.29645	1.30862e-2	144.03538		Vanillic acid
11.913	1	VB	33.40659	2.91058e-3	1.84742		Caffeic acid
12.472	1	BV	59.90018	7.37702e-3	8.39581		Syringic acid
13.300	1		-	-	-		Vanillin
15.000	1		-	-	-		p- Coumaric acid
16.400	1		-	-	-		Ferulic acid
17.557	1	BV	8.94304	9.01166e-2	15.31242		Benzoic acid
17.863	1	VB	196.12918	3.75734e-2	140.01570		Rutin
18.570	1	BB	28.45583	1.94033e-1	104.90624		Ellagic
19.540	1	BV	86.11564	2.69247e-3	4.40542		o- Coumaric acid
20.019	1	BV	1287.48596	3.15794e-2	772.50265		Salicylic acid
21.586	1	VV	254.79634	1.10285e-1	533.90220		Myricetin
24.500	1		-	-	-		Cinnamic acid
25.249	1	BB	6.51041	5.67785e-3	7.02337e-1		Quercitin
25.800	1		-	-	-		rosemarinic
26.500	1		-	-	-		Neringein
27.469	1	VV	92.54826	6.39900e-2	112.52111		Kampherol

Totals : 1852.39008

2 Warnings or Errors :

Warning : Calibration warnings (see calibration table listing)
Warning : Calibrated compound(s) not found

===

*** End of Report ***
